# Therapeutically targeting head and neck squamous cell carcinoma through synergistic inhibition of LSD1 and JMJD3 by TCP and GSK-J1

**DOI:** 10.1038/s41416-019-0680-6

**Published:** 2019-12-18

**Authors:** Wei Zhang, Jie Cheng, Pengfei Diao, Dongmiao Wang, Wei Zhang, Hongbing Jiang, Yanling Wang

**Affiliations:** 1 Jiangsu Key Laboratory of Oral Disease, Nanjing Medical University, 210029 Nanjing, P. R. China; 2 Department of Oral and Maxillofacial Surgery, Affiliated Stomatological Hospital, Nanjing Medical University, 210029 Nanjing, P. R. China; 3 Department of Oral Pathology, Affiliated Stomatological Hospital, Nanjing Medical University, 210029 Nanjing, P. R. China

**Keywords:** Head and neck cancer, Drug development

## Abstract

**Background:**

The histone demethylase LSD1 is a key mediator driving tumorigenesis, which holds potential as a promising therapeutic target. However, treatment with LSD1 inhibitors alone failed to result in complete cancer regression.

**Methods:**

The synergistic effects of TCP (a LSD1 inhibitor) and GSK-J1 (a JMJD3 inhibitor) against HNSCC were determined in vitro and in preclinical animal models. Genes modulated by chemical agents or siRNAs in HNSCC cells were identified by RNA-seq and further functionally interrogated by bioinformatics approach. Integrative siRNA-mediated gene knockdown, rescue experiment and ChIP-qPCR assays were utilised to characterise the mediators underlying the therapeutic effects conferred by TCP and GSK-J1.

**Results:**

Treatment with TCP and GSK-J1 impaired cell proliferation, induced apoptosis and senescence in vitro, which were largely recapitulated by simultaneous LSD1 and JMJD3 knockdown. Combinational treatment inhibited tumour growth and progression in vivo. Differentially expressed genes modulated by TCP and GSK-J1 were significantly enriched in cell proliferation, apoptosis and cancer-related pathways. SPP1 was identified as the mediator of synergy underlying the pro-apoptosis effects conferred by TCP and GSK-J1. Co-upregulation of LSD1 and JMJD3 associated with worse prognosis in patients with HNSCC.

**Conclusions:**

Our findings revealed a novel therapeutic strategy of simultaneous LSD1 and JMJD3 inhibition against HNSCC.

## Background

Head and neck squamous cell carcinoma (HNSCC) represents one of the most common malignancies worldwide with more than 35,000 cancer-related deaths per year, thus posing a great clinical challenge.^1^ Comprehensive and multimodality therapeutic approaches, including surgical ablation, chemotherapy and radiotherapy against HNSCC, have been established for decades.^2^ Nevertheless, its long-term survival rate has not been significantly improved, especially for those with advanced diseases. High incidence of locoregional recurrence, cervical lymph node metastasis and therapeutic resistance remarkably contribute to therapeutic failure and mortality.^3^ Great efforts have been devoted to delineating the genetic and molecular mechanisms driving HNSCC initiation and progression.^4^ However, such efforts and progress have modest beneficial effects on development of novel therapeutic approaches. Therefore, identifying druggable targets for HNSCC has been an imperative need to improve the therapeutic efficiency and patients’ survival.

Aberrant epigenetic dysregulations, including DNA methylation and histone modifications, have been established as hallmarks of cancer and valuable therapeutic targets.^5^ These epigenetic ‘reader, modifier and easer’ have been identified as oncogenes or tumour-suppressor genes involved in cancer initiation, progression and therapeutic failure.^6^ Importantly, the inherent reversible nature of these epigenetic alterations enables the epigenetic modulators as promising therapeutic targets against cancer.^7^ Among these cancer-relevant epigenetic modifiers, the lysine-specific demethylase 1 (LSD1, also known as KDM1A), a key component of various transcriptional co-repressor complexes such as CoREST (co-repressor for element-1-silencing transcriptional factor) and NuRD (nucleosome remodelling and histone deacetylation), selectively removes the methyl from H3K4me1/2 and in turn mediates gene repression.^8,9^ It has also been identified as a bona fide oncogene overexpressed across a broad spectrum of malignancies, and as a druggable target with translational potentials.^10–13^ Genetic depletion or pharmacological inhibition of LSD1 potently inhibited cell migration, epithelial-to-mesenchymal transition, chemoresistance and cancer stem cell properties, and sometimes stimulated antitumour immunity, ultimately restraining cancer growth and metastasis.^13–17^ We and others have reported that evaluated LSD1 promotes cell proliferation, cell cycle progression, invasion as well as chemosensitivity in HNSCC.^10,13,17^ Moreover, pharmacological inhibition of LSD1 by tranylcypromine (TCP) markedly inhibited tumour overgrowth in vivo, thus suggesting LSD1 as a promising druggable target for HNSCC.^13^ However, single TCP treatment failed to result in tumour regression, which might be associated with its relatively low potency at low dosage.^13,15^ However, TCP analogues with higher potency induced severe toxicity in vivo at the concentration wherein it attenuated the AML burden by LSD1 inhibition.^14,15^ Considering these facts, rationally designed combinational approach based on TCP and other anticancer drugs might be a viable and reasonable strategy to enhance therapeutic potency and reduce unwanted side effects.^14,18^

In addition to LSD1, JMJD3 (Jumonji domain containing-3, also known as KDM6B), another cancer-related epigenetic modulator, demethylates H3K27me3 to H3K27me2 or H3K27me1, leading to transcriptional activation of target genes.^19^ Several lines of evidence have shown that aberrant upregulation or downregulation of JMJD3 was observed in diverse types of cancer, suggesting a possible context-dependent expression of JMJD3.^20^ For example, the expression of JMJD3 was significantly decreased in renal cell carcinoma and colorectal cancer, while it was elevated in gastric cancer, bladder cancer and hepatocellular carcinoma.^21–24^ Noticeably, pioneering works have developed small-molecule chemical agents GSK-J1/J4 to potently inhibit the H3K27me3 demethylation activities of JMJD3 and reduce cancer overgrowth in multiple models.^25–27^ These findings suggest that therapeutic targeting of JMJD3 might be feasible and effective in some cancer contexts. However, the expression of JMJD3 and its roles in HNSCC remain incompletely known until now.

Previous reports have revealed that intricate crosstalk or functional interplay between histone-modifying enzymes contributes to tissue haemostasis and diseases including cancer, thus implying that combinational delivery of epigenetic compounds might be a viable approach with more potency.^28–31^ For instance, LSD1 interacted with JMJD2C and cooperatively stimulated androgen receptor-dependent gene transcription by removing methyl groups from mono-, di- and trimethylated H3K9 in prostate carcinoma.^31^ In this study, we sought to identify a synergetic approach based on TCP and other epigenetic compounds to inhibit HNSCC. Our findings revealed that combinational treatment with TCP and GSK-J1 had synergetic therapeutic effects against HNSCC in in vitro and in vivo preclinical models. This synergetic effect might be attributed to the transcriptional changes of genes involving cell proliferation and apoptosis following chemical treatment. SPP1 (secreted phosphoprotein 1, encoding osteopontin, OPN) was identified as one of the mediators involved in pro-apoptotic functions upon TCP and GSK-J1 exposure.

## Methods

All experimental details, including cellular experiments, animal studies and bioinformatics analyses, are provided in Supplementary Materials and Methods, and Supplementary Tables 1–6.

### Cell lines, epigenetic compounds, siRNAs and DNA constructs

Detailed information regarding cell lines, chemical compounds, siRNAs or vectors is provided in Supplementary Materials and Methods section. Information about siRNA sequences and antibodies is shown in Supplementary Tables 2 and 3.

### Drug combination screening in vitro

Each drug was arranged in 96-well plates, yielding a concentration equal to IC_50_ of a given drug before screening. For screening, ~3000 cells per well were seeded in the 96-well plates, and then treated with a single compound or with a combination of TCP and each epigenetic compound for another 72 h. Cell viability was measured using the CCK-8 assay. Combination index (CI) and fraction-affected (Fa) values were calculated using Compusyn software.

### CCK-8, colony formation and cell apoptosis by flow cytometry and β-galactosidase staining

CCK-8, colony formation assay, cell apoptosis by flow cytometry and β-galactosidase staining were performed as reported previously.^13,32^

### Genome-wide RNA sequence and bioinformatics analyses

Cal27 cells were treated with chemicals or siRNAs as indicated for 48 h. Two biological replicates per condition were performed. Total RNA was extracted and purified using oligo (dT)-attached magnetic beads. The RNA library was generated by PCR reactions and further sequenced by a BGISEQ-500 sequencer (BGI, China). Gene expression was quantified by RSEM software and further compared by NOISeq software. These differentially expressed genes were further assessed by GO, KEGG and gene set enrichment analysis (GSEA, version 3.0).

### 4-nitroquinoline 1-oxide (4NQO)-induced and xenograft HNSCC models and drug treatment

Six-week-old female C57BL/6 mice and female nude mice (BALB/c-nu) with similar weight were purchased from the Animal Center of Nanjing Medical University and housed in the animal room of the SPF laboratory under standard conditions. Both 4NQO-induced and xenograft HNSCC models were established as described previously.^33–35^ In 4NQO-induced model, initiation of drug treatment at this time point was chosen because previous reports have shown that following 16 weeks of 4NQO administration and another 6–10 weeks, mice developed identifiable SCC on the tongue.^33,34^ This schedule for drug administration was based on our prior work and other reports.^13,18,27^ TCP was administered 5 days per week at a dose of 10 mg/kg animal weight, while GSK-J1 was administered concomitantly at a dose of 25 mg/kg animal weight. In the xenograft model, mice-bearing xenograft tumours were randomly divided into four subgroups (at least six mice per group), which were scheduled to receive the following treatments: 10 mg/kg TCP, 25 mg/kg GSK-J1 or a combination of both agents and vehicle as control. These treatments were performed for 5 days a week for 3 consecutive weeks. The animal body weight and tumour volume were measured twice a week. The final tumour volume and weight were measured when mice were killed. All animals were euthanised by CO_2_ asphyxiation and cervical dislocation, and then tumour samples were harvested and processed for H&E and immunohistochemical staining. All animal experiments were reviewed and approved by Animal Experimental Committees of Nanjing Medical University and performed in accordance with institutional animal welfare guidelines. All animal studies were reported according to the ARRIVE guidelines for reporting experiments involving animals.

### Statistical analyses

All quantitative data were presented as mean ± SD from two or three independent experiments and compared with Student's *t* test or ANOVA with Bonferroni post hoc test unless otherwise specified. Synergy or additivity was calculated by CI method for combinations of multiple doses of drugs. Synergism is defined as a more than additive effect (CI < 1). Patient survival was estimated using Kaplan–Meier method and compared with log-rank test. *P* values less than 0.05 (two-sided) were considered statistically significant. All statistical analyses were performed using GraphPad Prism 8 or SPSS 21.0 software.

## Results

### Chemical combination screen identifies GSK-J1 acting synergistically with TCP in HNSCC cells

**Fig. 1:**
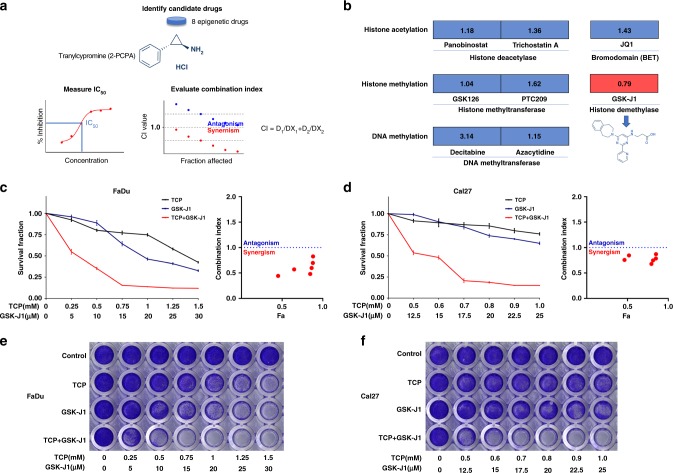
Drug combination screen identifies GSK-J1 acting synergistically with TCP. **a** The workflow of drug combination screen. IC_50_, median inhibitory concentration. CI, combination index. **b** Eight compounds targeting five classes of epigenetic modulators were examined in a combination screen with TCP in FaDu cells. The CI quantitatively depicts synergism (CI < 1), additive effect (CI = 1) and antagonism (CI > 1). **c**, **d** Sensitivity of FaDu, Cal27 to TCP alone, GSK-J1 alone or TCP combined with GSK-J1. Survival fraction (left) and the CI (right) are shown for each of these two cell lines. Fa, fraction affected. Error bars represent means ± SD. **e**, **f** Crystal violet staining of anchorage-dependent colony formation assay indicates the sensitivity of cells to control (DMSO), TCP, GSK-J1 or TCP combined with GSK-J1. Effect of treatments is shown for FaDu and Cal27 cells.

Recently, we and others have offered evidence that LSD1 inhibition by TCP or genetic knockdown has promising anticancer effects in diverse cancer contexts including HNSCC.^13,15,16^ However, considering its low efficiency as monotherapy, TCP in combination with other anticancer chemicals might be synergistic to achieve more potent effects. Given the crosstalk and functional cooperation among diverse epigenetic modifiers,^28–30^ we here sought to identify the epigenetic chemicals that can work in synergy with TCP in HNSCC. To address this, as illustrated in Fig.1a, we initiated a drug combination screen in vitro using TCP and other eight well-characterised epigenetic compounds that have been established as potent anticancer therapeutics.^7^ The IC_50_ values for each chemical agent in FaDu cells are listed in Supplementary Table 1. As shown in Fig. 1b, c and Supplementary Fig. 1, data from the average combination index (CI) values of TCP and eight chemicals revealed that only GSK-J1 had robust synergistic effects with TCP. Complementarily, the results from colony formation assay collaborated this idea regarding the synergistic effect between TCP and GSK-J1 in HNSCC cells (Fig. 1e). Consistently, strong synergistic effects of TCP with GSK-J1 were also observed in two other HNSCC cells (Cal27 and HN6) (Fig. 1d, f and Supplementary Fig. 2). Moreover, we employed another JMJD3 inhibitor GSK-J4 and found similar results concerning the synergistic inhibitory effects between TCP and GSK-J4 in HNSCC (Supplementary Fig. 3). Notably, we also tested this drug combination in four cancerous cell lines beyond HNSCC, human bone mesenchymal stem cells (BMSCs) and adipose-derived stem cells (ADSCs). As shown in Supplementary Fig. 4, this synergy between TCP and GSK-J1 was also observed in these malignant cells examined. However, we failed to find this synergy in both BMSCs and ADSCs, thus favouring the selective effects of this drug combination on cancerous cells. Collectively, our in vitro experimental findings indicated synergistic anticancer effects of TCP and GSK-J1 in HNSCC.

### TCP and GSK-J1 synergistically induced cell apoptosis and senescence in HNSCC cells

**Fig. 2:**
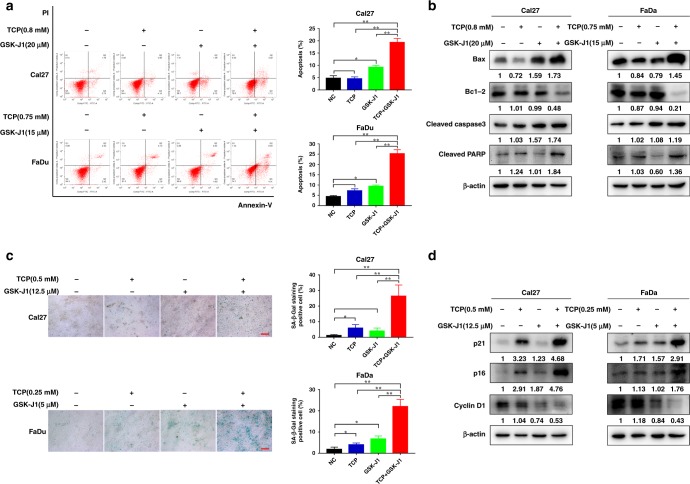
Combinational TCP and GSK-J1 treatment synergistically induces prominent cell apoptosis and senescence in vitro. **a** Percentages of apoptotic cells in Cal27 and FaDu cells following treatment with TCP and GSK-J1 alone or in combination (48 h) were measured by Annexin V/PI assay. **b** The protein levels of these apoptotic markers after treatments as indicated were determined by western blot. The concentrations of TCP and GSK-J1 are 0.8 and 0.75 mM, 20 and 15 μM in Cal27 and FaDu, respectively. **c** SA-β-gal staining (left panel) and percentages of senescent cells (right quantitative bar graph) were measured by senescence β-galactosidase cell-staining assay after 48 h of drug treatment. **d** The protein levels of these senescence-related markers after treatments as indicated were measured by western blot. The concentrations of TCP and GSK-J1 are 0.5 and 0.25 mM, 12.5 and 5 μM in Cal27 and FaDu, respectively. Data shown here are mean ± SD from three independent experiments, *P < 0.05, **P < 0.01, ANOVA analyses with Tukey's multiple comparisons test.

Induction of cells undergoing apoptosis and senescence represents the pivotal therapeutic effect executed by epigenetic chemicals in diverse cancers.^5^ Thus, we wondered whether cell apoptosis and/or senescence were responsible for these observed synergistic effects of TCP and GSK-J1 in HNSCC. To resolve this, we exposed both Cal27 and FaDu cells with TCP and GSK-J1 alone or in combination, and then determined cell apoptosis and senescence. As shown in Fig. 2a, combinational treatment with TCP and GSK-J1 significantly resulted in much more cells undergoing apoptosis than single agent alone. The results from flow cytometry showed that TCP and GSK-J1 exposure in Cal27 cells induced 20.1% of apoptotic cells, while single agent resulted in 4.5% and 9.2% of apoptotic cells, respectively. Next, we measured the abundance of apoptosis-relevant markers such as pro-apoptotic Bax, anti-apoptotic Bcl-2, cleaved caspase-3 and cleaved PARP in cells treated with these agents. Significant upregulation of Bax, cleaved caspase-3 and cleaved PARP, and downregulation of Bcl-2 were found in cells treated with both agents as compared with cells treated with single agent alone (Fig. 2b). Moreover, as shown in Fig. 2c, combinational exposure of TCP and GSK-J1 in Cal27 induced more positive SA-β-gal staining cells compared with single agent alone. The percentages of SA-β-gal-positive cells treated with TCP and GSK-J1 alone were 5.9% and 4.1%, whereas TCP and GSK-J1 combination induced 26.4% SA-β-gal-positive cells. In line with this phenomenon, combination treatment led to more abundance of p16 and p21, the markers of cell senescence, as well as downregulation of cyclin D1 (Fig. 2d). Taken together, our findings revealed that TCP and GSK-J1 synergistically induced cell apoptosis and senescence in HNSCC cells.

### Synergistic effect of TCP and GSK-J1 might result from LSD1 and JMJD3 inhibition in HNSCC

**Fig. 3:**
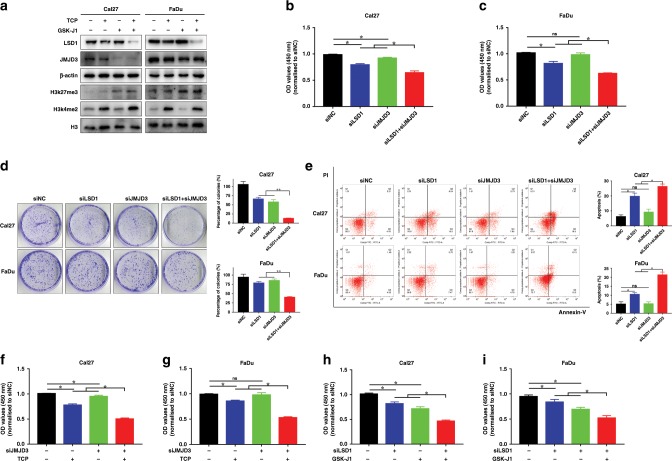
siRNA-mediated LSD1 and JMJD3 knockdown synergistically inhibits cell proliferation and induces cell apoptosis in vitro. **a** The protein levels of LSD1, JMJD3 and H3K4me2, H3K27me3 following TCP and JMJD3 treatment, were determined by western blot. Total H3 and β-actin were used as loading controls, respectively. **b**, **c** Cell proliferation was measured by CCK-8 viability assay in Cal27 and FaDu cells transfected with siRNAs as indicated for 48 h. **d** The potentials and quantifications of colony formation were significantly inhibited in Cal27 and FaDu cells transfected with siRNAs as indicated. **e** Increased percentages of apoptotic cells were evident in cells transfected with both LSD1 and JMJD3 targeting siRNAs. Cell apoptosis was assayed via Annexin V–PI staining. **f**, **g** JMJD3 knockdown sensitised cells to TCP treatment in vitro. Cell proliferation was measured when cells were initially transfected with siJMJD3 and then treated with TCP (0.8 mM in Cal27 and 0.75 mM in FaDu) by CCK-8 assay. **h**, **i** LSD1 knockdown sensitised cells to GSK-J1 treatment in vitro. Cell proliferation was measured when cells were initially transfected with siJMJD3 and then treated with GSK-J1 (20 μM in Cal27 and 15 μM in FaDu) by CCK-8 assay. Data shown here are mean ± SD from three independent experiments, *P < 0.05, ANOVA analyses with Tukey's multiple comparisons test.

Having revealed the synergistic effect of TCP and GSK-J1 in HNSCC cells, we next sought to determine whether LSD1 and JMJD3 targeted by these two chemicals were responsible for this synergy. As shown in Fig. 3a, treatment with TCP and GSK-J1, either alone or in combination, resulted in both LSD1 and JMJD3 downregulation. These results were in line with previous reports wherein TCP exposure reduced LSD1 protein expression in oesophageal carcinoma, breast cancer and neuroblastoma,^36,37^ and GSK-J4 treatment resulted in decreased JMJD3 abundance in breast cancer and glioma.^38,39^ As expected, global increase in H3K4me2 and H3K27me3, the primary targets modified by LSD1 and JMJD3, was observed. Next, we utilised the siRNA-mediated knockdown approach to determine the phenotypic changes in cells with LSD1 and JMJD3 knockdown. Two independent siRNAs targeting LSD1 and JMJD3 were designed and their knockdown efficiencies were verified. The siRNAs with more potency were selected for further analyses (Supplementary Fig. 5). As shown in Fig. 3b–d, combinational LSD1 and JMJD3 targeting siRNA treatment induced more pronounced inhibitory effects on cell proliferation, as measured by CCK-8 and colony formation assays. In addition, both siLSD1 and siJMJD3 induced more apoptotic cells as compared with single siRNA-treated cells (Fig. 3e). Furthermore, when cells were initially treated with siJMJD3 for 24 h and then followed with TCP exposure for another 48 h, more potent inhibition of cell proliferation was observed as compared with single treatment (Fig. 3f, g). On the other hand, when cells were initially transfected with siLSD1 and then treated with GSK-J1/J4 (Fig. 3h, i and Supplementary Fig. 6), cell proliferation was substantially repressed in cells treated with siLSD1 and GSK-J1/J4 as compared with those with single treatment. These results suggested that LSD1 knockdown enhanced the chemosensitivity to JMJD3 inhibitor in HNSCC cells, and vice versa. Together, these data suggest that synergistic anticancer effects of TCP and GSK-J1 might result from combinational inhibition of LSD1 and JMJD3 in HNSCC.

### Combinational treatment of TCP and GSK-J1 synergistically impaired tumour growth and progression in vivo

**Fig. 4:**
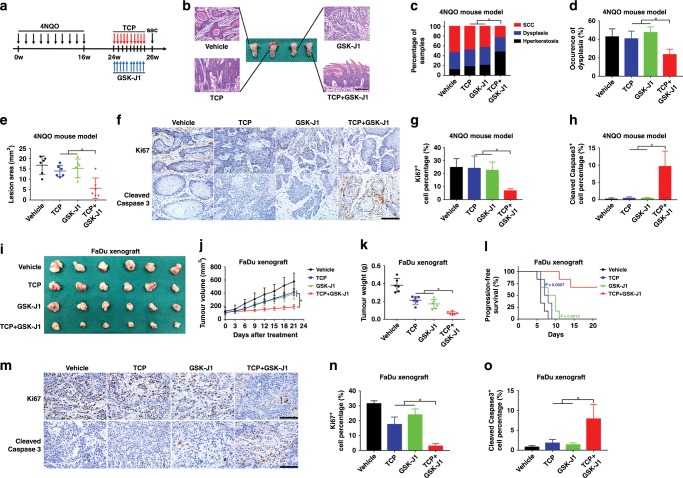
Combinational TCP and GSK-J1 administration inhibits tumour growth and progression in a preclinical model. **a** Schematic diagram showing the experimental protocol for combinational therapy in 4NQO-induced HNSCC model. Mice were randomly divided into four subgroups as follows: vehicle, TCP, GSK-J1 and TCP plus GSK-J1. TCP was administered 5 days per week via i.p. at 10 mg/kg. GSK-J1 was administered 5 days per week via i.p. at 25 mg/kg. **b** Representative H&E staining of SCC in tongue from different subgroups. Scale bar: 100 μm. **c** The proportions of hyperkeratosis, dysplasia and SCC in all mouse tongue specimens to evaluate the effect of drugs on the progress of HNSCC. Quantification of pathological lesions in different subgroups (*n* = 6). **d** Quantification of the incidence of dysplasia in different subgroups (*n* = 6). **e** Quantification of lesion areas from different subgroups (*n* = 6). **f** Immunostaining of Ki67 and cleaved caspase-3 in tongue SCC treated with TCP and GSK-J1 alone or in combination as indicated. Scale bar: 100 μm. **g**, **h** Quantification of Ki67 (**f**) and cleaved caspase-3- (**g**) positive staining cells in samples treated with TCP, GSK-J1 alone or in combination. **i** Tumour samples were harvested from HNSCC xenograft model treated with TCP, GSK-J1 alone or in combination. TCP was administered 5 days per week via i.p. at 10 mg/kg. GSK-J1 was administered 5 days per week via i.p. at 25 mg/kg. **j** Tumour volume was monitored in FaDu xenograft mice during the whole drug treatment. **k** Tumour weight was measured when animals were killed (*n* = 6). **l** Cumulative incidence plot depicting the percentage of tumours in each treatment group that has doubled in volume as a function of time. *P* values were calculated with log-rank test. **m** Representative staining of Ki67 and cleaved caspase-3 in samples after different treatments were shown. Scale bar: 100 μm. **n**, **o** Quantifications of Ki67^+^ (**n**) and cleaved caspase-3^+^ (**o**) staining cells protein expression were shown. Data shown here are mean ± SD from three independent experiments, *P < 0.05, **P < 0.01, ANOVA analyses with Tukey's multiple comparisons test.

To determine the therapeutic efficacy of combinational treatment of TCP and GSK-J1 in vivo, we utilised two preclinical HNSCC models, including 4NQO-induced model and xenograft model. In the 4NQO-induced HNSCC model, mice were randomly grouped and received intraperitoneal injection of TCP, GSK-J1 or TCP plus GSK-J1, respectively. As illustrated in Fig. 4a, after 16 weeks of 4NQO exposure and another 8 weeks, mice-bearing tongue SCC were treated with TCP, GSK-J1 or their combination 5 days a week for consecutive 2 weeks and then euthanised for sample harvest. As shown in Fig. 4b, c, histopathological examinations of tongue samples revealed that TCP plus GSK-J1 significantly inhibited HNSCC formation, as evidenced by much less incidence of invasive SCC found in animals treated with both agents as compared with animals treated with single agent alone. The incidence of dysplasia in animals treated with both agents was the lowest, suggesting that combinational administration of these agents blocked disease progression in vivo (Fig. 4d). In addition, TCP plus GSK-J1 robustly reduced surface areas of the lesions, whereas single agent had only partial effects (Fig. 4e). Immunohistochemical staining of proliferative marker Ki67 and apoptotic marker cleaved caspase-3 revealed much less Ki67-positive staining cells and more cleaved caspase-3 staining cells in samples derived from animals treated with combinational agents relative to samples treated with single agent (Fig. 4f–h).

To further determine the synergistic effect of TCP and GSK-J1, we develop a HNSCC xenograft model in which FaDu cells were subcutaneously inoculated into blanks of immunodeficient nude mice. In this xenograft model, mice-bearing tumours were randomly assigned into four groups to receive TCP, GSK-J1 or a combination of TCP and GSK-J1, and vehicle, respectively. As shown in Fig. 4i, j, treatment with TCP or GSK-J1 alone modestly reduced tumour volume, whereas their combination significantly inhibited tumour growth, as evidenced by marginally increased volume of tumours in animals treated with both agents. After intraperitoneal drug administration for 3 weeks, mice were killed and tumour xenograft was harvested and weighted. Compared with the vehicle-treated samples, treatment with single agent or both significantly decreased tumour weight. Noticeably, combinational treatment markedly decreased tumour weight as compared with single-agent treatment (Fig. 4k). Moreover, combinational treatment also resulted in significantly higher progression-free survival as compared with single treatment (Fig. 4l). As shown in Fig. 4m–o, samples from animals receiving combinational treatment had the lowest amount of Ki67^+^ staining cells and the highest amount of cleaved caspase-3^+^ cells, whereas samples from animals treated with TCP or GSK-J1 alone had modest changes in Ki67 and cleaved caspase-3 staining as compared with vehicle-treated control. Together, these results from two preclinical animal models provide compelling evidence that TCP and GSK-J1 treatment synergistically inhibited HNSCC tumour growth and progression in vivo by inhibiting cell proliferation and inducing cell apoptosis.

### Synergistic anticancer effects of TCP and GSK-J1 might be due to transcriptional modulation of genes involved in cell proliferation and apoptosis

**Fig. 5:**
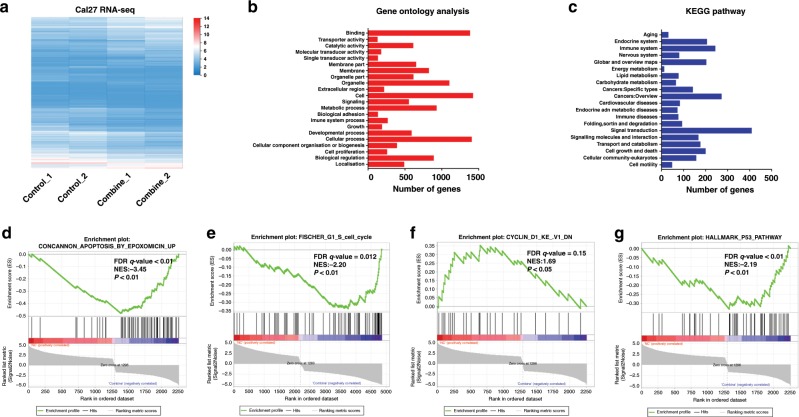
Combinational TCP and GSK-J1 treatment induces hundreds of differentially expressed genes enriched in cancer-associated biological events and pathways. **a** The heatmaps showing the differentially expressed genes by genome-wide transcriptional sequencing after TCP and GSK-J1 treatment in Cal27 cells. *n* = 2 biological replicates for each condition. **b**, **c** Gene ontology (**b**) and KEGG pathway (**c**) analyses of those differentially expressed genes revealed significant enrichment in cancer-related biological categories and pathways. **d**–**g** Gene set enrichment analysis (GSEA) of those differentially expressed genes revealed significant enrichment in apoptosis, cell cycle, cyclin D1 and p53 pathway.

To delineate the molecular mechanisms underlying the synergistic anticancer roles of TCP and GSK-J1 in HNSCC cells, Cal27 cells treated with both agents were subjected to genome-wide RNA-seq assay. As shown in Fig. 5a, a total number of 2258 genes were significantly changed with more than twofold upon TCP and GSK-J1 exposure. Among them, 962 genes were upregulated, while 1296 genes were downregulated. Subsequently, these differentially expressed genes (DEGs) were subjected to bioinformatics assays to unravel the potential biological roles of TCP and GSK-J1 responsible for their therapeutic effects. As shown in Fig. 5b, Gene Ontology (GO) analyses indicated that these DEGs were significantly enriched in cell proliferation and growth categories. In addition, KEGG pathway analyses indicated that these DEGs were highly enriched in categories, including cell growth and death as well as cancer (Fig. 5c). Consistently, the results from gene set enrichment analysis (GSEA) showed that combinational treatment significantly modulated genes involved in cell cycle, apoptosis, cyclin D1 and p53 pathways, which were largely in agreement with our findings from in vitro and in vivo experiments (Fig. 5d–g). In addition, as shown in Supplementary Fig. 7A, B, these DEGs were significantly enriched in tumorigenesis of nasopharyngeal carcinoma and breast cancer. Furthermore, we monitored the genome-wide transcriptional changes in Cal27 cells treated with both siRNAs targeting LSD1 and JMJD3. As shown in Supplementary Fig. 8A, 2244 DEGs were detected with 678 upregulated and 1566 downregulated. Consistent with the data in cells treated with both agents (Fig. 5a–g), these DEGs were significantly enriched in categories, including cell proliferation, growth, cell cycle and apoptosis (Supplementary Fig. 8B–D). Moreover, as anticipated, 1042 overlapped genes (~46%) were found in DEGs derived from cells exposed to either two agents or siRNAs (Supplementary Fig. 8E), which supported the general concordance between chemical and siRNA treatments (hypergeometric *P* < 0.0001). Taken together, these findings supported that combinational treatment with TCP and GSK-J1 resulted in potent anticancer effects presumably by transcriptionally regulating genes involved in cell proliferation and apoptosis in HNSCC.

### Identification of SPP1 as one of the mediators involved in pro-apoptotic roles of TCP and GSK-J1

**Fig. 6:**
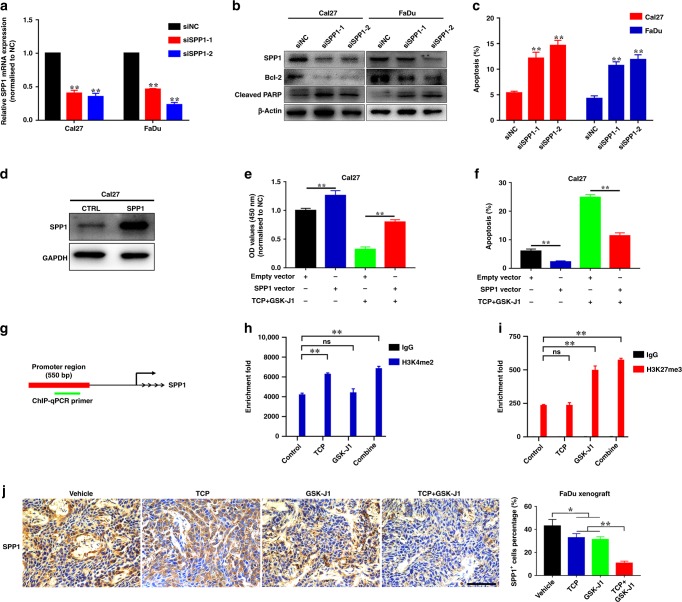
Identification of SPP1 as the mediator involved in the pro-apoptotic roles of TCP and GSK-J1. **a** The mRNA levels of SPP1 were measured by qRT-PCR when siRNAs targeting SPP1 were introduced into Cal27 and FaDu cells for 24 h. **b** The protein levels of SPP1 and apoptotic markers were determined by western blot when Cal27 and FaDu were treated with siRNAs targeting SPP1. Non-targeting siRNA was utilised as negative control (siNC). **c** Increased percentages of cells undergoing apoptosis were evident following SPP1 knockdown as assayed by Annexin V–PI staining. **d** Enforced overexpression of SPP1 was confirmed in Cal27 with SPP1-stable overexpression. **e** Enforced SPP1 overexpression attenuated the impaired cell proliferation induced by TCP plus GSK-J1 treatment in Cal27 cells. Cell proliferation was measured in Cal27 cells treated with either SPP1- overexpressing plasmid alone or plasmid followed by TCP plus GSK-J1 (TCP: 0.8 mM, GSK-J1: 20 μM). **f** Enforced SPP1 overexpression attenuated the pro-apoptosis induced by TCP plus GSK-J1 treatment in Cal27 cells. Cell apoptosis was measured by Annexin V–PI staining assay in Cal27 cells treated with either SPP1-overexpressing plasmid alone or plasmid followed by TCP plus GSK-J1 (TCP: 0.8 mM, GSK-J1: 20 μM). **g** Schematic diagram of the promoter region of SPP1 (–2329 to –1780 bp) harbouring H3K4me2 and H3K27me3 marks. The ChIP-qPCR primers were designed to amplify this region in immunoprecipitated DNA. **h**, **i** The enrichment changes in H3K4me2 and H3K27me3 at the SPP1 promoter region were assessed by ChIP-qPCR assays after drug treatment (alone or combined, 48 h). IgG was used as negative control. **j** Representative staining and quantifications of SPP1^+^ staining in samples after different treatments were shown. Scale bar: 100 μm. Data shown here are mean ± SD from three independent experiments, *P < 0.05, **P < 0.01, ANOVA analyses with Tukey's multiple comparisons test.

To explore the mediators of the synergy between TCP and GSK-J1, we focused on the apoptotic pathway. As shown in Supplementary Fig. 9A, 67 apoptosis-associated genes were significantly changed with more than twofold based on GSEA analyses. These genes were filtered in terms of their individual expression and its effects on survival in TCGA–HNSCC cohort. Three apoptosis-associated genes, SPP1, SLC3A2 and HSPH1, were significantly upregulated and their upregulations associated with unfavourable prognosis in HNSCC (Supplementary Fig. 9B–D). Interestingly, we found that SPP1 was also among the top 30 genes downregulated by TCP and GSK-J1 exposure (Supplementary Fig. 9E, F). Moreover, qRT-PCR assays confirmed that TCP and GSK-J1, or siRNAs targeting LSD1 plus JMJD3, synergistically reduced SPP1 mRNA expression in Cal27 cells (Supplementary Fig. 9G, H). Indeed, SPP1 has been identified as a key oncogene involved in apoptosis and associated with cancer prognosis.^40,41^ Due to the lack of knowledge regarding SPP1 in HNSCC, we next determine its roles via siRNA-mediated loss-of-function approach. Following SPP1 knockdown in vitro, cell proliferation was significantly impaired (Fig. 6a, b and Supplementary Fig. 9I, J). Flow cytometric assay revealed increased proportions of apoptotic cells in siSPP1-treated cells (from 5.34% to 14.63% in Cal27, from 4.26% to 11.88% in FaDu, Fig. 6c and Supplementary Fig. 9K). In parallel, significant upregulation of cleaved caspase-3 and downregulation of Bcl-2 were found in siSPP1-treated cells (Fig.6B). To further validate the SPP1 as a mediator involved in apoptosis after drug treatment, we utilised in vitro rescue experiment and ChIP-qPCR assay. As shown in Fig. 6d–f and Supplementary Fig. 9L, enforced overexpression of SPP1 significantly attenuated the effects of TCP and GSK-J1 on cell proliferation and apoptosis in Cal27 cells. We mapped both H3K4me2 and H3K27me3 enrichment marks upstream of SPP1 TSS (transcriptional start site) region in multiple cell lines using online Cistrome database and identified the region spanning 550 bp enriched by H3K4me2 and H3K27me3 marks (Supplementary Fig. 10A, B and Fig. 6g). As shown in Fig. 6h, i, the H3K4me2 and H3K27me3 enrichments were significantly increased in these regions upon TCP and GSK-J1 treatment, which in turn impaired SPP1 transcription. Consistently, immunohistochemical staining in samples harvested from xenograft experiments revealed significantly fewer SPP1^+^ cells in samples derived from animals receiving both TCP and GSK-J1 as compared with samples treated with single agents (Fig. 6j). Collectively, these findings strongly supported that TCP and GSK-J1 treatment increased the enrichment of H3K4me2 and H3K27me3 in SPP1 promoter region, in turn epigenetically impairing its transcription and inducing cells undergoing apoptosis in HNSCC.

### Risk-score formula development and validation using online-available profiling data

Inspired by the transcriptional changes upon TCP and GSK-J1 treatment, we hypothesised that these DEGs might have prognostic significance in HNSCC. To address this, we developed a novel prognostic score comprising four genes (HOXB9, SPNS3, FGD3 and SC5D) from these 2258 DEGs by sequential univariate regression analysis, robust likelihood-based modelling and multivariate regression analysis using TCGA–HNSCC dataset as training cohort. This risk score was calculated by the following formula: risk score = HOXB9 × 0.12774 + SPNS3 × (–0.01175) + FGD3 × (–0.37110) + SC5D × 0.27629. Consistently, these genes have been found to be significantly associated with prognosis in several human cancers, including gastric, breast and colon cancer.^42–44^ In addition, HOXB9 has been identified as a putative oncogene promoting cell proliferation, migration, metastasis and cancer stem cell self-renewal.^42,45^ The optimal cutoff value for this score was 0.953 derived from the ROC curve using TCGA–HNSCC dataset (Supplementary Fig. 11A). Kaplan–Meier plots indicated that patients in the high-risk subgroup had markedly reduced survival as compared with those with low risk in the TCGA–HNSCC dataset (*P* < 0.0001, Supplementary Fig. 11B). Furthermore, two other independent HNSCC cohorts (GSE41613 and GSE42743)^46^ were further utilised as testing and validation cohorts to verify the prognostic utility of this risk score. Consistently, this risk score robustly stratified patients into subgroups with high or low survival ratios in both testing and validation cohorts (*P* = 0.026, 0.00088; log-rank test; Supplementary Fig. 11C, D).

### LSD1 and JMJD3 expression has a prognostic impact on patients with HNSCC

Previous reports have documented that aberrant LSD1 overexpression significantly associated with aggressiveness and unfavourable prognosis in cancer.^10,13,36,37^ However, the expression pattern and prognostic significance of JMJD3 in HNSCC remain largely underexplored. We next explored mutational and expression patterns of LSD1 and JMJD3 in HNSCC using the TCGA–HNSCC dataset. As shown in Supplementary Fig. 12A, B, LSD1 mRNA was significantly upregulated in a fraction of HNSCC samples as compared with their non-tumour counterparts (*P* < 0.0001), whereas JMJD3 mRNA expression in HNSCC was comparable to their non-tumour counterparts (*P* = 0.4764). Rare genetic alterations of LSD1 in HNSCC are unlikely to be responsible for its overexpression (Supplementary Fig. 12C). However, gain and amplification of LSD1 or JMJD3 might account for their upregulations in selected samples (Supplementary Fig. 12D, E).

Given the synergy between TCP and GSK-J1 in HNSCC, we wondered whether integrative examination of LSD1 and JMJD3 expression might improve prognostic prediction. We utilised another HNSCC cohort (GSE41613) and plotted the Kaplan–Meier and ROC curves. As shown in Supplementary Fig. 13A, B, we found that LSD1 or JMJD3 mRNA expression can stratify patients into subgroups with low or high survival in GSE41613 dataset when the optimal cutoff values derived from the ROC curve were utilised (*P* = 0.012, *P* = 0.023, log-rank test). Positive correlation between LSD1 and JMJD3 expression was also found in GSE41613 dataset (Supplementary Fig. 13C). Finally, as shown in Supplementary Fig. 13D, the Kaplan–Meier analyses indicated that patients in both high LSD1 plus JMJD3 subgroup had the worst prognosis among all subgroups (*P* = 0.0046, log-rank test). Univariate and multivariate Cox regression assays identified combined LSD1/JMJD3 overexpression as an independent prognostic factor after adjusting some demographic and clinical parameters (Supplementary Table 7).

## Discussion

The past few decades have witnessed tremendous translational significance of aberrant epigenetic dysregulation underlying tumorigenesis and epigenetic modifiers exploited as targets against cancer.^5^ Here we integrate the findings from in vitro experiments, in vivo animal models and bioinformatics analyses and reveal that TCP works synergistically with GSK-J1 against HNSCC by modulating genes involved in cell proliferation and apoptosis. Our study offers a strong rationale for clinical application of LSD1 inhibitors in combination with JMJD3 inhibitors for patients with HNSCC.

Several lines of evidence have revealed that LSD1 is an essential oncogene driving cancer initiation, overgrowth and metastatic dissemination in multiple contexts including HNSCC.^13,15,16^ Our results together with others have provided clues that genetic or pharmacological depletion of LSD1 induces therapeutic effects in HNSCC.^12,13,17^ However, the potency of single-agent chemotherapy usually seems limited, as evidenced by the facts such as initially effective but acquired therapeutic resistance and toxicities when used in high dosage. Findings from clinical applications and animal studies have revealed that side effects and toxicities of LSD1 inhibitors, like monoamine oxidase inhibitors (MAOI), include orthostatic hypotension, dizziness, drowsiness, insomnia and nausea in human, as well as seizures and high mortality in animals.^37,47,48^ These above-mentioned challenges necessitated the explorations to design therapeutic agents or strategies to targeting LSD1 more effectively with fewer toxicities. Here we employed a small-scale in vitro drug combination screen and identified GSK-J1 as a potent, synergistic compound in combination with TCP against HNSCC. Their synergistic effects were confirmed in two preclinical animal models. Interestingly, previous studies have identified synergistic combination of LSD1 inhibitors and histone deacetylase (HDAC) inhibitors in multiple cancers.^49,50^ However, we failed to find this combination although TCP was individually testified with two common HDACi inhibitors (Trichostatin A, Panobinostat) in HNSCC. We speculate that it might be due to diverse genetic background and aetiologic factors between HNSCC and other malignancies. These anticancer effects induced by TCP and GSK-J1 were similar as phenotypical changes following siRNA-mediated knockdown of LSD1 and JMJD3. Of note, our data indicated that reduced abundance of LSD1 and JMJD3 was observed in cells treated with TCP and GSK-J1, respectively. Similar phenomenon was also observed in selected cancers, albeit not common in all cancers, thus suggesting cancer-type difference.^36–39^ Thus, we believe that therapeutic effects exerted by TCP and GSK-J1 probably result from LSD1 and JMJD3 inhibition, not only their activities but also their expression in HNSCC. Of course, given the targeting specificity of GSK-J1 and TCP,^51^ we here cannot rule out the possibility that other mediators beyond LSD1 and JMJD3 might also account for therapeutic effects of TCP and GSK-J1 in HNSCC.

With regard to the therapeutic effects induced by TCP and GSK-J1, our results indicated that impaired cell proliferation and increased cell apoptosis and senescence were largely responsible. Noticeably, combinational treatment of TCP and GSK-J1 not only induced tumour shrinkage, but also impaired HNSCC development and progression, as evidenced by markedly reduced tumour volume in the xenograft model and decreased proportions of dysplasia and SCC in the 4NQO-induced model. Indeed, these findings were conceivable and consistent with previous results concerning the biological functions of LSD1 and JMJD3 in diverse cancer contexts.^13,17,21,52^ For example, integrated genomic analyses identified E2F signalling as a key mediator of LSD1 in promoting cell proliferation in oral cancer.^17^ Depletion of LSD1 or JMJD3 by genetic and pharmacological approaches significantly reduced cell proliferation and triggered cells undergoing apoptosis in oral cancer, small-cell lung carcinoma and breast cancer.^13,53,54^ In addition, both LSD1 and JMJD3 were intricately involved in cellular senescence.^55,56^ In support of these, our GSEA analyses of genes affected by TCP and GSK-J1 revealed significant enrichment in cell cycle, apoptosis, ageing and p53 pathway. On the other hand, previous reports have also reported that LSD1 and JMJD3 facilitate cell migration and invasion, EMT as well as chemoresistance in select cancer contexts. Targeted inhibition of LSD1 or JMJD3 robustly impaired these above-mentioned malignant properties.^15,16,23^ Thus, it remains an interesting issue concerning whether combinational treatment with TCP and GSK-J1 also induces these therapeutic effects beyond cell proliferation, apoptosis and senescence in HNSCC. Taken together, our findings revealed that TCP and GSK-J1 synergistically restrained tumour growth by inducing anti-proliferative, pro-apoptotic and pro-senescence effects.

Previous findings have established that LSD1 and JMJD3 as histone-modifying enzymes execute their functions largely via demethylation of histone H3 lysines 4, 9 or 27, resulting in activation or inactivation of gene expression.^19,57–59^ Consistent with these findings,^58,59^ our RNA-seq revealed hundreds of DEGs following TCP and GSK-J1 treatment and their enrichments in multiple cancer-related biological categories and pathways. Here we filtered these DEGs and identified SPP1 as the mediator underlying the pro-apoptotic effects conferred by TCP and GSK-J1 by siRNA-mediated knockdown, rescue experiments as well as ChIP-qPCR assays (Fig. 6 and Supplementary Fig. 9, 10). Our results supported the model that TCP and GSK-J1 exposure increased H3K4me2 and H3K27me3 enrichments in SPP1 promoter region, in turn inhibiting its transcription and then inducing cell apoptosis. These findings were well consistent with recent reports concerning SPP1 as anti-apoptotic genes underlying several human cancers.^40,60^

Given the essential roles of LSD1 in HNSCC tumorigenesis and potent therapeutic effects induced by TCP and GSK-J1, we developed and validated a prognostic risk score based on transcriptional profiling data following TCP and GSK-J1 by statistical and bioinformatics approaches. Intriguingly, this risk score robustly stratified patients into subgroups with high or low survival by interrogation of mRNA expression of four genes with specificity and sensitivity. Consistent with their prognostic significance in HNSCC, these genes have been found to be significantly associated with prognosis in several human cancers, including gastric, breast and colon cancer.^42–44^ However, besides HOXB9, the biological functions of other three genes in human cancer remain largely underexplored and deserve further investigations. Furthermore, our bioinformatics analyses revealed that not only higher expression of LSD1 or JMJD3 significantly associated with reduced survival, but also combined high expression of LSD1 and GSK-J1 indicated the worst prognosis in patients with HNSCC. Collectively, these findings suggest that determination of LSD1 and JMJD3 expression or their downstream targets might provide useful prognostic information.

There are some limitations and unresolved questions concerning our findings. Although our data revealed the SPP1 as one of molecular mediators underlying the pro-apoptotic effects of concurrent LSD1 and JMJD3 pharmacological inhibition, the detailed molecular mechanisms underlying the synergy between TCP and GSK-J1 in HNSCC remain to be further explored. Moreover, the effective predictive biomarkers for patient selection and prognostic prediction for this combinational therapeutic strategy are still to be identified before the translation of this strategy in the clinic. In addition, the proposed risk score is needed to further validate in prospectively collected cohorts with both HPV-negative and -positive HNSCC.

In conclusion, our data reveal potent and synergistic therapeutic effects of LSD1 inhibitor TCP and JMJD3 inhibitor GSK-J1 in HNSCC. These findings offer ample evidence to support combinational targeting of LSD1 and JMJD3 as a novel promising strategy for HNSCC.

## Supplementary information

Supplementary Files for BJC TH-2019-4116R1

## Data Availability

All original data are available upon reasonable requestAll original data are available upon reasonable request.

## References

[1] Siegel RL, Miller KD, Jemal A (2018). Cancer statistics, 2018. CA Cancer J. Clin..

[2] Santuray RT, Johnson DE, Grandis JR (2018). New therapies in head and neck cancer. Trends Cancer.

[3] Ringash J (2015). Survivorship and quality of life in head and neck cancer. J. Clin. Oncol..

[4] Cancer Genome Atlas N. (2015). Comprehensive genomic characterization of head and neck squamous cell carcinomas. Nature.

[5] Dawson MA, Kouzarides T (2012). Cancer epigenetics: from mechanism to therapy. Cell.

[6] Campbell RM, Tummino PJ (2014). Cancer epigenetics drug discovery and development: the challenge of hitting the mark. J. Clin. Invest..

[7] Bennett RL, Licht JD (2018). Targeting epigenetics in cancer. Annu. Rev. Pharmacol. Toxicol..

[8] Shi Y, Lan F, Matson C, Mulligan P, Whetstine JR, Cole PA (2004). Histone demethylation mediated by the nuclear amine oxidase homolog LSD1. Cell.

[9] Lee MG, Wynder C, Cooch N, Shiekhattar R (2005). An essential role for CoREST in nucleosomal histone 3 lysine 4 demethylation. Nature.

[10] Yuan C, Li Z, Qi B, Zhang W, Cheng J, Wang Y (2015). High expression of the histone demethylase LSD1 associates with cancer cell proliferation and unfavorable prognosis in tongue cancer. J. Oral. Pathol. Med..

[11] Hayami S, Kelly JD, Cho HS, Yoshimatsu M, Unoki M, Tsunoda T (2011). Overexpression of LSD1 contributes to human carcinogenesis through chromatin regulation in various cancers. Int. J. Cancer.

[12] Hoshino I, Akutsu Y, Murakami K, Akanuma N, Isozaki Y, Maruyama T (2016). Histone demethylase LSD1 inhibitors prevent cell growth by regulating gene expression in esophageal squamous cell carcinoma cells. Ann. Surg. Oncol..

[13] Wang Y, Zhu Y, Wang Q, Hu H, Li Z, Wang D (2016). The histone demethylase LSD1 is a novel oncogene and therapeutic target in oral cancer. Cancer Lett..

[14] Schenk T, Chen WC, Gollner S, Howell L, Jin L, Hebestreit K (2012). Inhibition of the LSD1 (KDM1A) demethylase reactivates the all-trans-retinoic acid differentiation pathway in acute myeloid leukemia. Nat. Med..

[15] Harris WJ, Huang X, Lynch JT, Spencer GJ, Hitchin JR, Li Y (2012). The histone demethylase KDM1A sustains the oncogenic potential of MLL-AF9 leukemia stem cells. Cancer Cell.

[16] McDonald OG, Wu H, Timp W, Doi A, Feinberg AP (2011). Genome-scale epigenetic reprogramming during epithelial-to-mesenchymal transition. Nat. Struct. Mol. Biol..

[17] Narayanan SP, Singh S, Gupta A, Yadav S, Singh SR, Shukla S (2015). Integrated genomic analyses identify KDM1A's role in cell proliferation via modulating E2F signaling activity and associate with poor clinical outcome in oral cancer. Cancer Lett..

[18] Singh MM, Johnson B, Venkatarayan A, Flores ER, Zhang J, Su X (2015). Preclinical activity of combined HDAC and KDM1A inhibition in glioblastoma. Neuro Oncol..

[19] Xiang Y, Zhu Z, Han G, Lin H, Xu L, Chen CD (2007). JMJD3 is a histone H3K27 demethylase. Cell Res..

[20] Burchfield JS, Li Q, Wang HY, Wang RF (2015). JMJD3 as an epigenetic regulator in development and disease. Int. J. Biochem. Cell Biol..

[21] Xu Z, Xia Y, Xiao Z, Jia Y, Li L, Jin Y (2019). Comprehensive profiling of JMJD3 in gastric cancer and its influence on patient survival. Sci. Rep..

[22] Tokunaga R, Sakamoto Y, Nakagawa S, Miyake K, Izumi D, Kosumi K (2016). The prognostic significance of histone lysine demethylase JMJD3/KDM6B in colorectal cancer. Ann. Surg. Oncol..

[23] Tang B, Qi G, Tang F, Yuan S, Wang Z, Liang X (2016). Aberrant JMJD3 expression upregulates slug to promote migration, invasion, and stem cell-like behaviors in hepatocellular carcinoma. Cancer Res..

[24] Hong Z, Li H, Li L, Wang W, Xu T (2017). Different expression patterns of histone H3K27 demethylases in renal cell carcinoma and bladder cancer. Cancer Biomark..

[25] Kruidenier L, Chung CW, Cheng Z, Liddle J, Che K, Joberty G (2012). A selective jumonji H3K27 demethylase inhibitor modulates the proinflammatory macrophage response. Nature.

[26] Lochmann Timothy L., Powell Krista M., Ham Jungoh, Floros Konstantinos V., Heisey Daniel A. R., Kurupi Richard I. J., Calbert Marissa L., Ghotra Maninderjit S., Greninger Patricia, Dozmorov Mikhail, Gowda Madhu, Souers Andrew J., Reynolds C. Patrick, Benes Cyril H., Faber Anthony C. (2018). Targeted inhibition of histone H3K27 demethylation is effective in high-risk neuroblastoma. Science Translational Medicine.

[27] Hashizume R, Andor N, Ihara Y, Lerner R, Gan H, Chen X (2014). Pharmacologic inhibition of histone demethylation as a therapy for pediatric brainstem glioma. Nat. Med..

[28] Lee MG, Wynder C, Bochar DA, Hakimi MA, Cooch N, Shiekhattar R (2006). Functional interplay between histone demethylase and deacetylase enzymes. Mol. Cell Biol..

[29] Yu Y, Schleich K, Yue B, Ji S, Lohneis P, Kemper K (2018). Targeting the senescence-overriding cooperative activity of structurally unrelated H3K9 demethylases in melanoma. Cancer Cell.

[30] Vasilatos SN, Katz TA, Oesterreich S, Wan Y, Davidson NE, Huang Y (2013). Crosstalk between lysine-specific demethylase 1 (LSD1) and histone deacetylases mediates antineoplastic efficacy of HDAC inhibitors in human breast cancer cells. Carcinogenesis.

[31] Wissmann M, Yin N, Muller JM, Greschik H, Fodor BD, Jenuwein T (2007). Cooperative demethylation by JMJD2C and LSD1 promotes androgen receptor-dependent gene expression. Nat. Cell Biol..

[32] Li Z, Wang Y, Zhu Y, Yuan C, Wang D, Zhang W (2015). The Hippo transducer TAZ promotes epithelial to mesenchymal transition and cancer stem cell maintenance in oral cancer. Mol. Oncol..

[33] Chen D, Wu M, Li Y, Chang I, Yuan Q, Ekimyan-Salvo M (2017). Targeting BMI1(+) cancer stem cells overcomes chemoresistance and inhibits metastases in squamous cell carcinoma. Cell Stem Cell.

[34] Vitale-Cross L, Molinolo AA, Martin D, Younis RH, Maruyama T, Patel V (2012). Metformin prevents the development of oral squamous cell carcinomas from carcinogen-induced premalignant lesions. Cancer Prev. Res. (Phila).

[35] Wu Y, Wang Y, Diao P, Zhang W, Li J, Ge H (2019). Therapeutic Targeting of BRD4 in Head Neck Squamous Cell Carcinoma. Theranostics.

[36] Lim S, Janzer A, Becker A, Zimmer A, Schule R, Buettner R (2010). Lysine-specific demethylase 1 (LSD1) is highly expressed in ER-negative breast cancers and a biomarker predicting aggressive biology. Carcinogenesis.

[37] Schulte JH, Lim S, Schramm A, Friedrichs N, Koster J, Versteeg R (2009). Lysine-specific demethylase 1 is strongly expressed in poorly differentiated neuroblastoma: implications for therapy. Cancer Res.

[38] Sui A, Xu Y, Li Y, Hu Q, Wang Z, Zhang H (2017). The pharmacological role of histone demethylase JMJD3 inhibitor GSK-J4 on glioma cells. Oncotarget.

[39] Yan N, Xu L, Wu X, Zhang L, Fei X, Cao Y (2017). GSKJ4, an H3K27me3 demethylase inhibitor, effectively suppresses the breast cancer stem cells. Exp. Cell Res..

[40] Zhao J, Dong L, Lu B, Wu G, Xu D, Chen J (2008). Down-regulation of osteopontin suppresses growth and metastasis of hepatocellular carcinoma via induction of apoptosis. Gastroenterology.

[41] Zeng B, Zhou M, Wu H, Xiong Z (2018). SPP1 promotes ovarian cancer progression via Integrin beta1/FAK/AKT signaling pathway. Onco. Targets Ther..

[42] Hayashida T, Takahashi F, Chiba N, Brachtel E, Takahashi M, Godin-Heymann N (2010). HOXB9, a gene overexpressed in breast cancer, promotes tumorigenicity and lung metastasis. Proc. Natl Acad. Sci. USA.

[43] Chang WC, Cheng WC, Cheng BH, Chen L, Ju LJ, Ou YJ (2018). Mitochondrial Acetyl-CoA Synthetase 3 is Biosignature of Gastric Cancer Progression. Cancer Med..

[44] Ou Yang TH, Cheng WY, Zheng T, Maurer MA, Anastassiou D (2014). Breast cancer prognostic biomarker using attractor metagenes and the FGD3-SUSD3 metagene. Cancer Epidemiol. Biomarkers Prev..

[45] Lin J, Zhang D, Fan Y, Chao Y, Chang J, Li N (2018). Regulation of cancer stem cell self-renewal by HOXB9 antagonizes endoplasmic reticulum stress-induced melanoma cell apoptosis via the miR-765-FOXA2 axis. J. Invest. Dermatol..

[46] Lohavanichbutr P, Mendez E, Holsinger FC, Rue TC, Zhang Y, Houck J (2013). A 13-gene signature prognostic of HPV-negative OSCC: discovery and external validation. Clin Cancer Res..

[47] Sun Q, Ding D, Liu X, Guo SW (2016). Tranylcypromine, a lysine-specific demethylase 1 (LSD1) inhibitor, suppresses lesion growth and improves generalized hyperalgesia in mouse with induced endometriosis. Reprod. Biol. Endocrinol..

[48] Fiedorowicz JG, Swartz KL (2004). The role of monoamine oxidase inhibitors in current psychiatric practice. J. Psychiatr. Pract..

[49] Fiskus W, Sharma S, Shah B, Portier BP, Devaraj SG, Liu K (2014). Highly effective combination of LSD1 (KDM1A) antagonist and pan-histone deacetylase inhibitor against human AML cells. Leukemia.

[50] Singh MM, Manton CA, Bhat KP, Tsai WW, Aldape K, Barton MC (2011). Inhibition of LSD1 sensitizes glioblastoma cells to histone deacetylase inhibitors. Neuro Oncol..

[51] Heinemann B, Nielsen JM, Hudlebusch HR, Lees MJ, Larsen DV, Boesen T (2014). Inhibition of demethylases by GSK-J1/J4. Nature.

[52] Arcipowski KM, Martinez CA, Ntziachristos P (2016). Histone demethylases in physiology and cancer: a tale of two enzymes, JMJD3 and UTX. Curr. Opin. Genet. Dev..

[53] Mohammad HP, Smitheman KN, Kamat CD, Soong D, Federowicz KE, Van Aller GS (2015). A DNA hypomethylation signature predicts antitumor activity of LSD1 inhibitors in SCLC. Cancer Cell.

[54] Svotelis A, Bianco S, Madore J, Huppe G, Nordell-Markovits A, Mes-Masson AM (2011). H3K27 demethylation by JMJD3 at a poised enhancer of anti-apoptotic gene BCL2 determines ERalpha ligand dependency. EMBO J..

[55] Barradas M, Anderton E, Acosta JC, Li S, Banito A, Rodriguez-Niedenfuhr M (2009). Histone demethylase JMJD3 contributes to epigenetic control of INK4a/ARF by oncogenic RAS. Genes Dev..

[56] Castex J, Willmann D, Kanouni T, Arrigoni L, Li Y, Friedrich M (2017). Inactivation of Lsd1 triggers senescence in trophoblast stem cells by induction of Sirt4. Cell Death Dis..

[57] Hojfeldt JW, Agger K, Helin K (2013). Histone lysine demethylases as targets for anticancer therapy. Nat. Rev. Drug Discov..

[58] Huang Y, Greene E, Murray Stewart T, Goodwin AC, Baylin SB, Woster PM (2007). Inhibition of lysine-specific demethylase 1 by polyamine analogues results in reexpression of aberrantly silenced genes. Proc. Natl Acad. Sci. USA.

[59] Chen S, Ma J, Wu F, Xiong LJ, Ma H, Xu W (2012). The histone H3 Lys 27 demethylase JMJD3 regulates gene expression by impacting transcriptional elongation. Genes Dev..

[60] Yan W, Qian C, Zhao P, Zhang J, Shi L, Qian J (2010). Expression pattern of osteopontin splice variants and its functions on cell apoptosis and invasion in glioma cells. Neuro Oncol..

