# Supplementary material for: Therapeutically targeting head and neck squamous cell carcinoma through synergistic inhibition of LSD1 and JMJD3 by TCP and GSK-J1
Source: Br J Cancer. 2019 Dec 18;122(4):528–38. doi: 10.1038/s41416-019-0680-6 (PMC7028736; doi:10.1038/s41416-019-0680-6)
Supplement: Supplementary file 1 — Supplementary Files for BJC TH-2019-4116R1 [file 41416_2019_680_MOESM1_ESM.docx]

**Supplementary Materials and Methods**

**Cell lines, epigenetic compound, siRNAs and DNA constructs**

Three human HNSCC cell lines including Cal27, FaDu and HN6 were used. Cal27 and FaDu were purchased from American Type Culture Collection (ATCC, Manassas, VA, USA), while HN6 cells were kindly gifted from Prof. Wantao Chen (Shanghai Jiaotong University). All cancerous cells were authenticated by short tandem repeat profiling at regular intervals and grown in DMEN/F12 (Invitrogen) media containing 10% FBS (Gibco) and 100 units/ml antibiotics in a humidified incubator with 5% CO_2_ at 37℃. Mycoplasma detection was routinely performed during the whole course of this study.

Ten epigenetic compounds were purchased from the following vendors as detailed in **Supplementary Table 1**. TCP was dissolved in PBS while other drugs were dissolved and stored in DMSO until use. In vitro experiments, final concentration of DMSO was less than 0.1%. Other chemical regents were purchased from Sigma-Aldrich unless otherwise stated.

Two independent small interference RNAs (siRNA) targeting human LSD1 and JMJD3 were designed and synthesized. The siRNA sequences were listed in **Supplementary Table 2**. These siRNAs were transiently transfected into cells with Lipofectamine 3000 (Invitrogen) at final concentration of 100nM unless otherwise specified. Knockdown efficiencies were verified by western blot following transfection. In some instances, cells were initially transfected with siRNAs for 24h and then treated with indicated chemicals for another 48h, which were harvested and subjected for further analyses.

The SPP1 overexpressing construct tagged with single FLAG was generated by inserting the human SPP1 full-length cDNA template into plasmid GV141 (GeneChem Inc. Shanghai, China). Following transfection with SPP1 overexpressing plasmid by Lipofectamine 2000, stable cell clones were selected by G418 (1 mg/ml, Sigma) for 2 weeks and then pooled for further experiments.

**Drug combination screening in vitro**

Each drug was arranged in 96-well plates and yielding a concentration equal to IC50 of a given drug before screening. For screening, approximately 3000 cells per well were seeded in the 96-well plates, and then treated with a single compound or with a combination of TCP and each epigenetic compound for another 72 hours. Cell viability was measured using the CCK-8 assay. Combination index (CI) and fraction affected (Fa) values were calculated using Compusyn software.

**CCK-8 and** **colony formation assay**

Cell viability was assessed by absorbance using CCK-8 cell viability assay (Cell Counting Kit-8, Dojindo, Japan) according to manufacturer’s instructions. Approximately 3×10^3^ cells per well were seeded in the 96-well plates, and then incubated in new growth media containing 10% CCK-8 reaction solution. After incubation for 2 hours, the absorbance was measured according to a spectrophotometer microplate reader (Multiskan MK3, Thermo) at a wavelength of 450 nm. For colony formation assay in Figure 1E-F, approximately 3×10^3^ cells per well were seeded in the 96-well plates, and then treated with DMSO, TCP, GSK-J1, or a combination of TCP and GSK-J1 for another 5 days. After fixation, colonies were stained with 0.5% crystal violet for 30 min and counted. For colony formation assay in Figure 3D, pretreated cells (1000 cells/well) were seeded in a 6-well plate and cultured at 37℃ for 10 days. After fixation, colonies were stained with crystal violet for 30 minutes and visualized under microscope, photographed, and counted.

**Cell apoptosis assessed by flow-cytometric assay**

Cells were treated with trypsin (Gbico) and resuspended as single-cell suspension, and then cells were stained with Annexin V: PI Apoptosis Detection Kit (BD Bioscience) and submitted to a FACS Calibur flow cytometer (BD Biosciences). Data were analyzed with CellQuest Pro software (BD Biosciences).

**Senescence β-galactosidase cell staining**

Senescence β-galactosidase cell staining (SA-β-gal) was performed with β-gal staining kit (Cell signaling, #9860) according to the manufacturer’s instructions. Briefly, cells were fixed in 2% formaldehyde /0.2%glutaraldehyde /PBS for 15 minutes at room temperature and stained using β-galactosidase staining solution at 37℃ overnight. Five randomly selected fields were chosen for quantification of SA-β-gal positive cells using inverted microscopy. The percentage of SA-β-gal positive cells was calculated using Image J software.

**RNA extraction, qRT-PCR and ChIP-qPCR assays**

Total RNA was extracted with Trizol reagent (Invitrogen) and then subjected to reverse transcription and PCR reactions using PrimeScript^TM^ RT-PCR kit (Takara) as described previously. Relative mRNA expression was quantified as compared to internal control GAPDH using comparative CT method. The primers were listed as follows: SPP1 (forward: CTCCATTGACTCGAACGACTC, reverse: CAGGTCTGCGAAACTTCTTAGAT) and GAPDH (forward: AGGTGAAGGTCGGAGTCAAC, reverse: AGTTGAGGTCAATGAAGGGG).

ChIP-qPCR assay was performed using EZ-ChIP^TM^ Chromatin Immunoprecipitation Kit (Millipore) according to the manufacturer’s protocol. In brief, cells were fixed and crosslinked with 1% formaldehyde for 10 minutes and lysed. The chromatin extracts were sonicated with Bioruptor, then incubated overnight with antibodies for H3K4me2 and H3K27me3 (Cell signaling, USA), or normal mouse IgG (Millipore). Immunoprecipitated DNA was analyzed by qPCR for detecting the enrichment of H3K4me2 and H3K27me3 in the promoter region of SSP1. The primers for ChIP-qPCR reactions to amplify the overlapped regions of H3K4me2 and H3K27me3 marks were forward: AGGATAGGTAGGCTGGGCGA; reverse: GACTGCTTGAGAGGGCTGCT.

**Western blot**

Cells were harvested and lysed in ice-clod cell lysis buffer containing protease inhibitor cocktail (Invitrogen). The same amount of protein samples was electrophoresed through 7-10% SDS-PAGE and transferred to PVDF membranes (Bio-Rad). Following 5% non-fat milk or BSA blocking, these membranes were incubated at 4℃ overnight with primary antibodies followed by incubation with horseradish peroxidase (HRP)-conjugated secondary antibodies. Detailed information regarding antibodies were displayed in **Supplementary Table 3**. Immunoreactive bands on the blots were detected by ECL chemiluminescence kit (Bio-Rad) and measured using Image J software.

**4-nitroquinoline 1-oxide (4NQO)-induced HNSCC animal model and drug treatment**

For the 4NQO-induced HNSCC animal model, 6-week-old C57BL/6 mice were treated with drinking water containing 50μg/mL 4NQO for consecutive 16 weeks and then given with normal water for another 8 weeks.^33, 34^ Lesions in tongue were visually inspected twice every week. From 24^th^ week, these animals were randomly received TCP, GSK-J1 alone or the combination of both chemicals by intraperitoneal injection (*n* = 6 per group) for consecutive 2 weeks. Initiation of drugs treatment at this time point was chosen because previous reports have shown that following 16 weeks of 4NQO administration and another 6-10 weeks, mice developed identifiable SCC in tongue.^33, 34^ This schedule for drug administration was based on our prior work and other reports.^13, 18, 27^ TCP dissolved in saline was administered 5 days per week at a dose of 10 mg/kg animal weight. GSK-J1 dissolved in DMSO was administered concomitantly at a dose of 25 mg/kg animal weight. Animals with vehicle injection were used as control. Finally, upon mice were sacrificed, total tongue was harvested and subjected to the gross examination measurement of lesions and further histopathological analyses. All experiments involving animal subjects were in accordance with the institutional animal welfare guidelines and approved by Institutional Animal Care and Use Committee of Nanjing Medical University.

**HNSCC xenograft model and drug treatment**

Six-week-old female nu/nu mice were obtained and maintained in a specific pathologic-free environment. Cancer cells suspended in total 100μL PBS and Matrigel (1:1) were inoculated subcutaneously on the single or both flanks (at least 6 animals per experimental group). Tumor incidence and growth were monitored after inoculation and tumor diameters were measured by calipers every 3 days after tumor masses were identified. For drug treatment experiments, 2×10^6^ viable FaDu cells were inoculated subcutaneously in nude mice and grown until tumors size was approximately 100 mm^3^. Mice bearing xenograft tumors were randomly divided into four subgroups (at least 6 mice per group) which were scheduled to receive the following treatments: 10 mg/kg TCP, 25 mg/kg GSK-J1, or combination of both agents and vehicle as control. These treatments were performed for 5 days a week for 3 consecutive weeks. The animal body weight and tumor volume (calculated as length × width × width/2) were measured twice a week. Finally, mice were sacrificed and final tumor volume and weight were measured upon tumor samples were harvested. Tumor samples were processed for H&E staining and immunohistochemical staining.

**Immunohistochemical staining**

Immunohistochemical staining was performed on 4μm-thick slides from formalin-fixed paraffin-embedded samples using routine procedures as we reported previously.^10^ Negative controls without primary antibody incubation were included. Primary antibodies were used: anti-Ki67 (1:250, M724029-2, Dako), anti-active-capsase3 (1:200, #9661, Cell signaling), anti-SPP1 (1:100, 25715-1-AP, Proteintech). The images were further visualized under fluorescence microscope and the positively stained cells were quantified using ImageJ software.

**Genome-wide RNA-sequence and bioinformatics analyses**

Cal27 cells were treated with TCP plus GSK-J1 or vehicle for 48 h. Two biological replicates per condition were performed. Total RNA was extracted and purified using oligo (dT) attached magnetic beads. The RNA library was generated by PCR reactions and further sequenced by a BGISEQ-500 sequencer (BGI, China). After reads filtration and mapping, individual gene expression was quantified by RSEM software and further compared by NOISeq software. These differentially expressed genes were further assessed by GO, KEGG and gene set enrichment analysis (GSEA, version 3.0) analyses.

**Risk score formula development and validation**

To identify prognostic significance of genes regulated by TCP and GSK-J1 in HNSCC, we developed a risk score formula including the candidates which were weighted by their estimated regression coefficients in the multivariable Cox regression analysis using TCGA-HNSCC dataset as the training set. Based on this formula, the risk score for each patient in the training set was calculated. A receiver operating characteristic (ROC) curve was plotted by using R with survival ROC package to identify the optimal cutoff point for risk score with maximal sensitivity and specificity. Survival difference between patients in the low-risk and high-risk subgroups stratified by the cutoff point was assessed by Kaplan-Meier analyses and Log-rank test. Furthermore, this risk score formula was further validated by fitting in the testing set (GSE41613, GSE42743).^46^ Detailed demographic and clinical information in TCGA-HNSCC, GSE41613 and GSE42743 was provided in **Supplementary Tables 4-6.**

**Statistical analyses**

All quantitative data were presented as mean ± SD from two or three independent experiments and compared with Student's *t*-test or ANOVA with Bonferroni post hoc test unless otherwise specified. Synergy or additivity was calculated by combination index (CI) method for combinations of multiple doses of drugs. Synergism is defined as a more than additive effect (CI < 1). Patient survival was estimated using Kaplan-Meier method and compared with Log-rank test. *P* values less than 0.05 (two-sided) were considered statistically significant. All statistical analyses were performed using GraphPad Prism 8 or SPSS 21.0 software.

**Reference**

10. Yuan C, Li Z, Qi B, Zhang W, Cheng J, Wang Y. High expression of the histone demethylase LSD1 associates with cancer cell proliferation and unfavorable prognosis in tongue cancer. J Oral Pathol Med 2015, **44**, 159-65.

13. Wang Y, Zhu Y, Wang Q, Hu H, Li Z, Wang D, et al. The histone demethylase LSD1 is a novel oncogene and therapeutic target in oral cancer. Cancer Lett 2016, **374**, 12-21.

18. Singh MM, Johnson B, Venkatarayan A, Flores ER, Zhang J, Su X, et al. Preclinical activity of combined HDAC and KDM1A inhibition in glioblastoma. Neuro Oncol 2015, **17**, 1463-73.

27. Hashizume R, Andor N, Ihara Y, Lerner R, Gan H, Chen X, et al. Pharmacologic inhibition of histone demethylation as a therapy for pediatric brainstem glioma. Nat Med 2014, **20**, 1394-6.

33. Chen D, Wu M, Li Y, Chang I, Yuan Q, Ekimyan-Salvo M, et al. Targeting BMI1(+) Cancer Stem Cells Overcomes Chemoresistance and Inhibits Metastases in Squamous Cell Carcinoma. Cell Stem Cell 2017, **20**, 621-34 e6.

34. Vitale-Cross L, Molinolo AA, Martin D, Younis RH, Maruyama T, Patel V, et al. Metformin prevents the development of oral squamous cell carcinomas from carcinogen-induced premalignant lesions. Cancer Prev Res (Phila) 2012, **5**, 562-73.

46. Lohavanichbutr P, Mendez E, Holsinger FC, Rue TC, Zhang Y, Houck J, et al. A 13-gene signature prognostic of HPV-negative OSCC: discovery and external validation. Clin Cancer Res 2013, **19**, 1197-203.

**Supplementary Figure legends**

**Figure S1.**

**A-H:** TCP and 8 well-characterized epigenetic drugs were chosen for the initial screen. Sensitivity of FaDu to TCP alone, epigenetic drug alone, or epigenetic drug combined with TCP. Survival fraction (left) and the CI (right) are shown for FaDu cell line.

**Figure S2.**

**A:** Sensitivity of HN6 to TCP, GSK-J1 alone, or in combination. Survival fraction (left) and the CI (right) are shown for HN6 cell lines. Fa, fraction affected. Error bars represent means ± SD.

**B:** Crystal violet staining of anchorage-dependent colony formation assay indicates the sensitivity of cells to control (DMSO), TCP, GSK-J1, or TCP combined with GSK-J1. Effect of treatments is shown for HN6 cells.

**Figure S3.**

**A, B:** Sensitivity of FaDu and Cal27 to TCP, GSK-J4 alone, or in combination. Survival fraction (left) and the CI (right) are shown for each of these two cell lines. Fa, fraction affected. Error bars represent means ± SD.

**Figure S4.**

**A-E:** Sensitivity of gastric (BSG823), cervical (HeLa), breast (MCP-7) and colon (HT-29) cancer cell lines to TCP, GSK-J1 alone, or in combination. Survival fraction (left) and the CI (right) are shown for these cell lines. Fa, fraction affected. Error bars represent means ± SD.

**F:** Sensitivity of BMSCs and ADSCs to TCP, GSK-J1 alone, or in combination. Survival fraction was shown.

**Figure S5.**

**A:** Endogenous LSD1 was efficiently silenced by siLSD1 in Cal27 and FaDu cells. Non-targeting siRNA was utilized as negative control (siNC).

**B:** Endogenous JMJD3 was efficiently silenced by siJMJD3 in Cal27 and FaDu cells. Non-targeting siRNA was utilized as negative control (siNC).

Representative images of western blot are shown from 3 independent experiments.

**Figure S6.**

**A, B:** siLSD1 sensitized HNSCC cells to GSK-J4 in vitro. Cell proliferation was measured in cells were initially transfected with siLSD1 and then treated with GSK-J4 (5μM in Cal27 and 2.5μM in FaDu). Data shown here are mean ± SD from three independent experiments, **P* < 0.05, ANOVA analyses with Tukey's multiple comparisons test.

**Figure S7.**

**A, B:** GSEA analyses revealed that DEGs by TCP plus GSK-J1 were significantly enriched in nasopharyngeal carcinoma (**A**) and breast cancer (**B**).

**Figure S8.**

**A:** The heatmap showed the DEGs by genome-wide RNA-seq when Cal27 cells were transfected with siNC, siLSD1, siJMJD3 and siLSD1 plus siJMJD3 respectively.

**B, C:** Gene ontology (**B**) and KEGG pathway (**C**) analyses of these DEGs revealed significant enrichments in cancer-related biological categories and pathways.

**D:** Gene set enrichment analysis (GSEA) of these DEGs revealed significant enrichments in apoptosis, cell cycle and p53 pathway.

**E:** Venn diagram displayed the numbers of overlapped genes modulated by TCP plus GSK-J1 and DEGs modulated by siLSD1+siJMJD3 in Cal27 cells.

**Figure S9.**

**A:** The heatmap showed the DEGs from apoptosis-related pathway identified by GSEA in Cal27 cells. N = 2 biological replicates for each condition.

**B-D:** Overall survival analyses of TCGA-HNSCC patients with high or low expression of SPP1, SLC3A2 and HSPH1 mRNA (median value as cutoff) were estimated by Kaplan-Meier method and compared with Log-rank test.

**E:** The heatmap showed the top 30 downregulated genes b after TCP and GSK-J1 treatment in Cal27 cells. N = 2 biological replicates for each condition.

**F:** Venn diagram displaying the overlapped gene between apoptosis-related DEGs and top 30 downregulated DEGs.

**G, H:** The mRNA levels of SPP1 were measured by qRT-PCR when Cal27 cells were treated with drugs or siRNAs at indicated times.

**I, J:** Cell proliferation was remarkably suppressed when endogenous SPP1 was silenced as measured by CCK-8 assay.

**K, L:** Percentages of cell undergoing apoptosis were determined by Annexin V-PI staining assay in Cal27 cell treated with either siRNA (**K**) or SPP1 overexpressing plasmid plus drugs (**L**).

Data shown here are mean ± SD from three independent experiments, ***P* < 0.01, ANOVA analyses with Tukey's multiple comparisons test.

**Figure S10.**

**A, B:** Gene tracks from Cistrome Data Browser (http://cistrome.org/db/#/) shown H3K4me2 and H3K27me3 marks at SPP1 promoter region in multiple cell lines include 293T and cancerous cells. Both H3K4me2 and H3K27me3 marks were largely overlapped and enriched in 550bp region highlighted by blue box. All tracks were visualized by UCSC Genome Browser (<http://genome.ucsc.edu/>).

**Figure S11.**

**A:** Receiver operating characteristics (ROC) analysis of sensitivity and specificity of the 4-gene signature based risk score identified from differentially expressed genes modulated by TCP and GSK-J1 using TCGA-HNSCC dataset as training cohort. The blue dot represented the optimal cut-off value.

**B:** The Kaplan-Meier analyses indicated that this risk score robustly stratified patients into subgroups with high or low survival ratios. *P* values were calculated with log-rank test.

**C, D:** Prognostic utility of this risk score was further validated in another two independent cohorts (GSE41613 and GSE42743) as testing and validation cohorts. *P* values were calculated with log-rank test.

**Figure S12.**

**A, B:** The mRNA levels of LSD1 and JMJD3 (log2-transformed) were compared between HNSCC samples and normal counterparts using TCGA-HNSCC dataset. ***P* < 0.01 by Student’s *t* test. NS: not significant.

**C:** Genetic alterations of LSD1 and JMJD3 were detected in TCGA-HNSCC dataset via cBioPortal platform (http://www.cbioportal.org).

**D:** The proportions of copy number variations in TCGA-HNSCC dataset were shown.

**E:** The mRNA expression of LSD1 and JMJD3 correlated with their copy number variations. Data obtained from the TCGA-HNSCC dataset. ***P* < 0.01, ANOVA analyses with Tukey's multiple comparisons test.

**Figure S13.**

**A:** Receiver operating characteristics (ROC) analysis identified the optimal cutoff value of LSD1 expression in the GSE41613 cohort which stratified patients into subgroups with high and low survival. *P* values were calculated with log-rank test.

**B:** ROC analysis identified the optimal cutoff value of JMJD3 in the GSE41613 cohort which stratified patients into subgroups with high and low survival. *P* values were calculated with log-rank test.

**C:** Correlation between LSD1 mRNA and JMJD3 mRNA was identified in GSE41613 cohort (*P*=0.0378, N = 96, Pearson’s correlation).

**D:** Patients with co-overexpression of LSD1 and JMJD3 had the worst prognosis in GSE41613 cohort as estimated by Kaplan-Meier method. *P* values were calculated with log-rank test.

Supplementary Figure 1


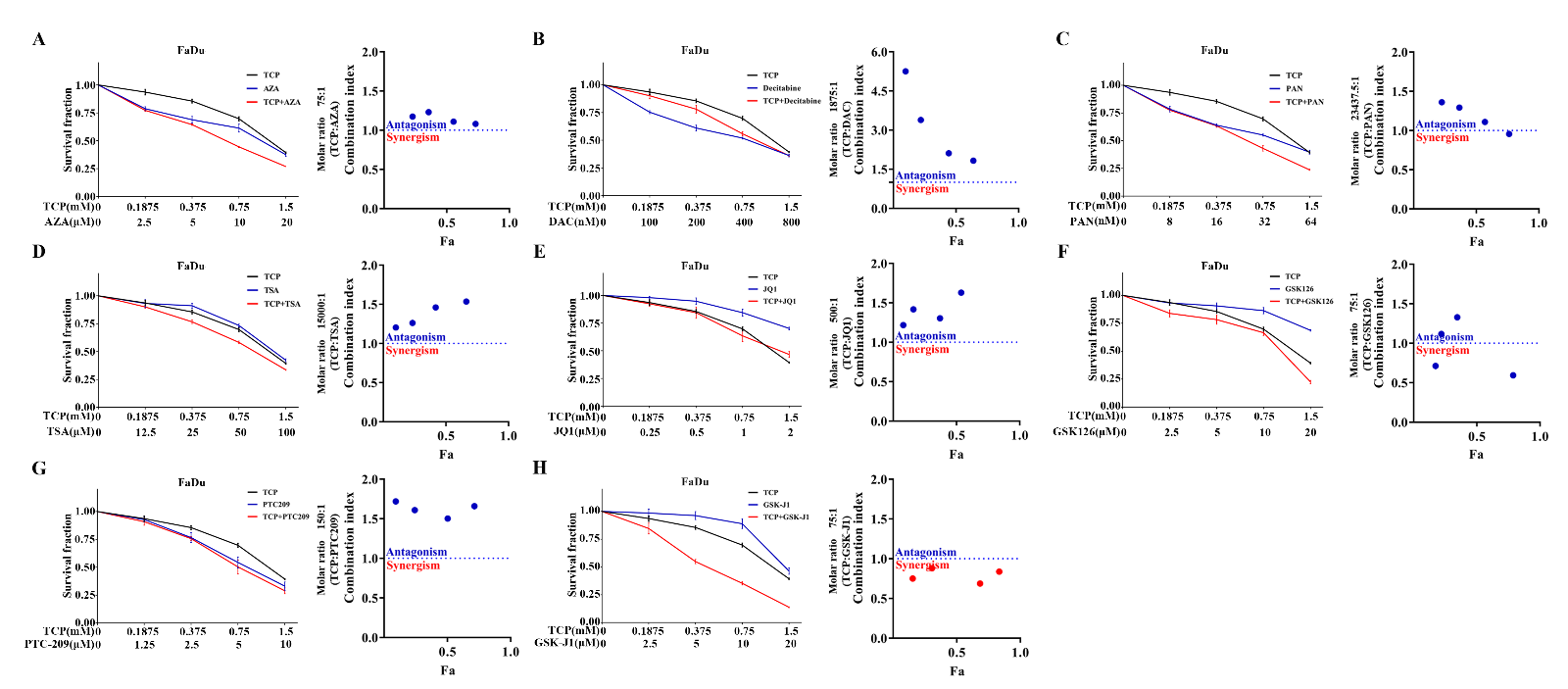


Supplementary Figure 2


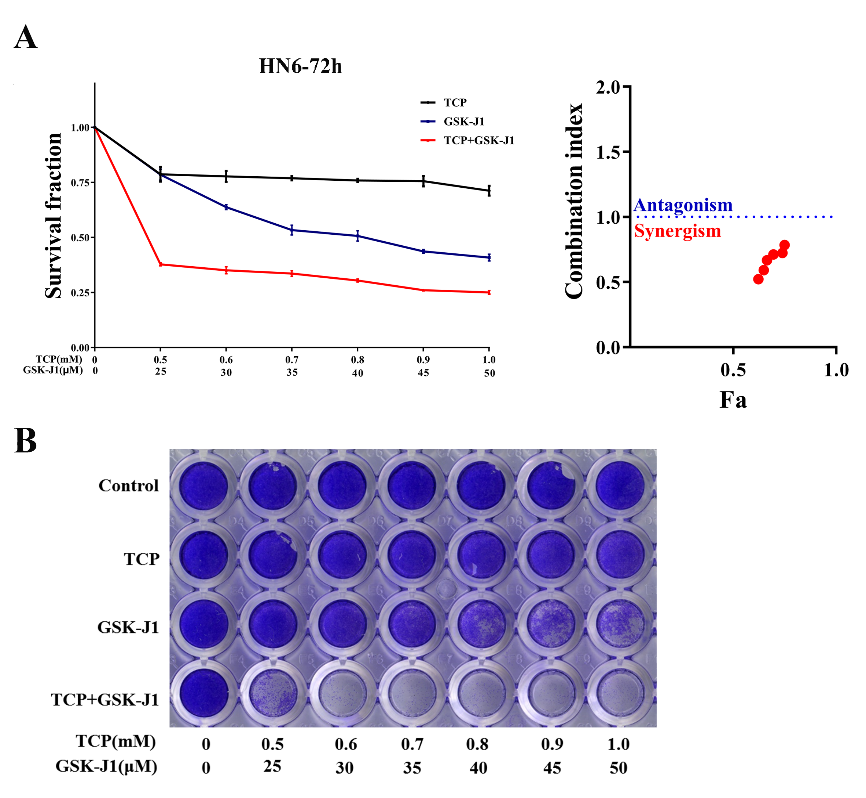


Supplementary Figure 3


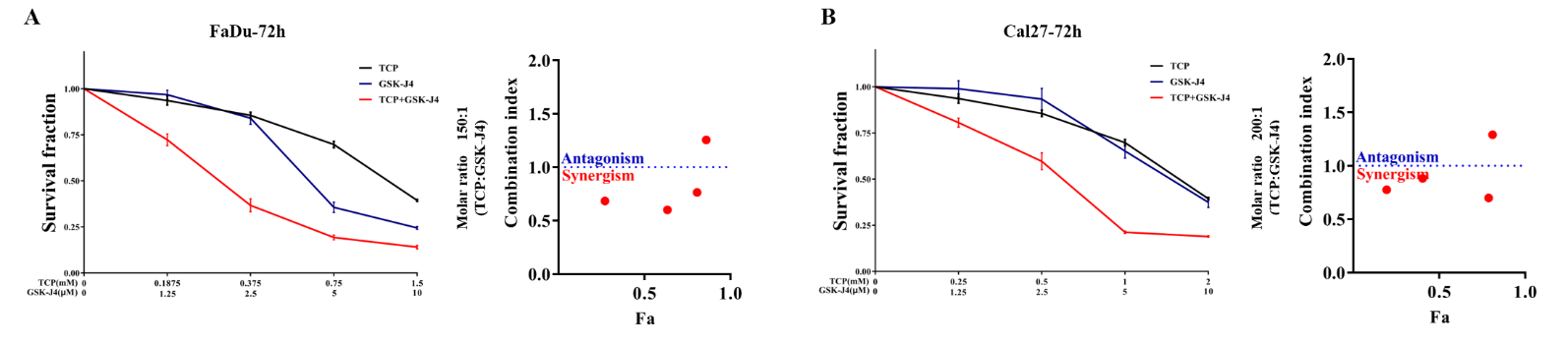


Supplementary Figure 4


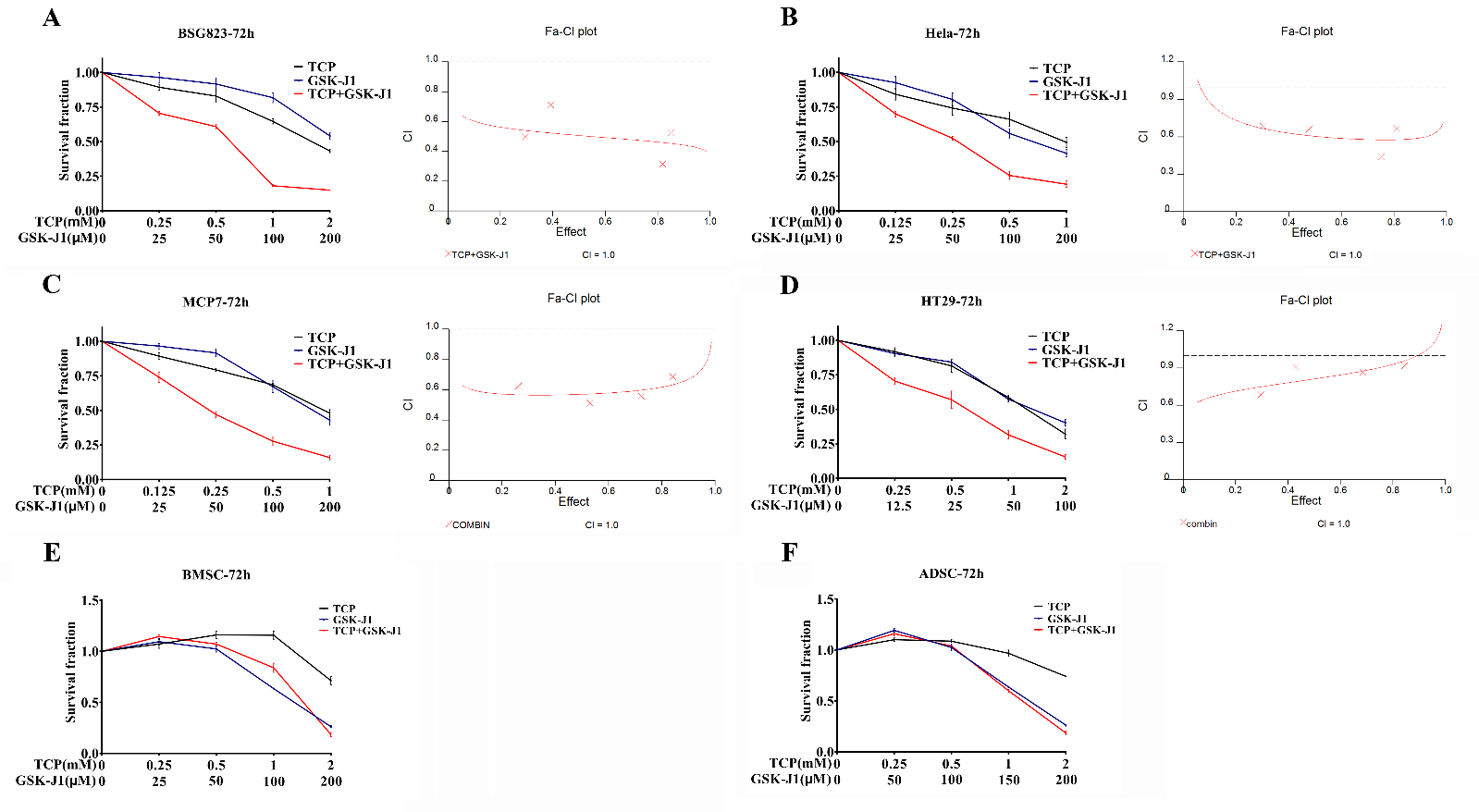


Supplementary Figure 5


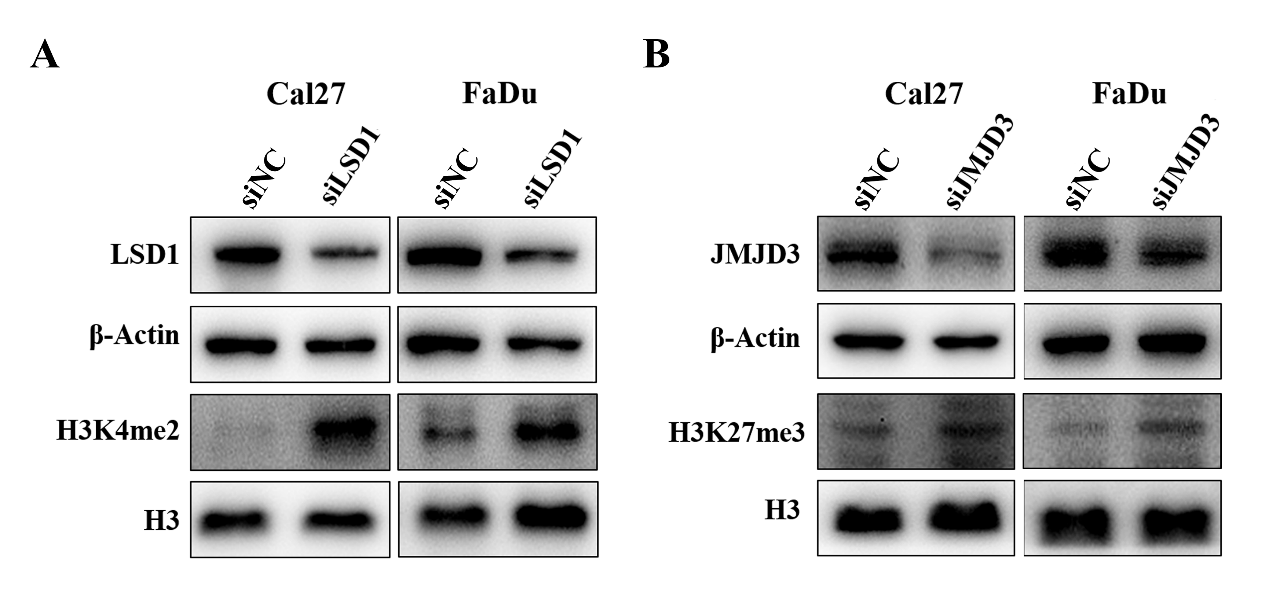


Supplementary Figure 6


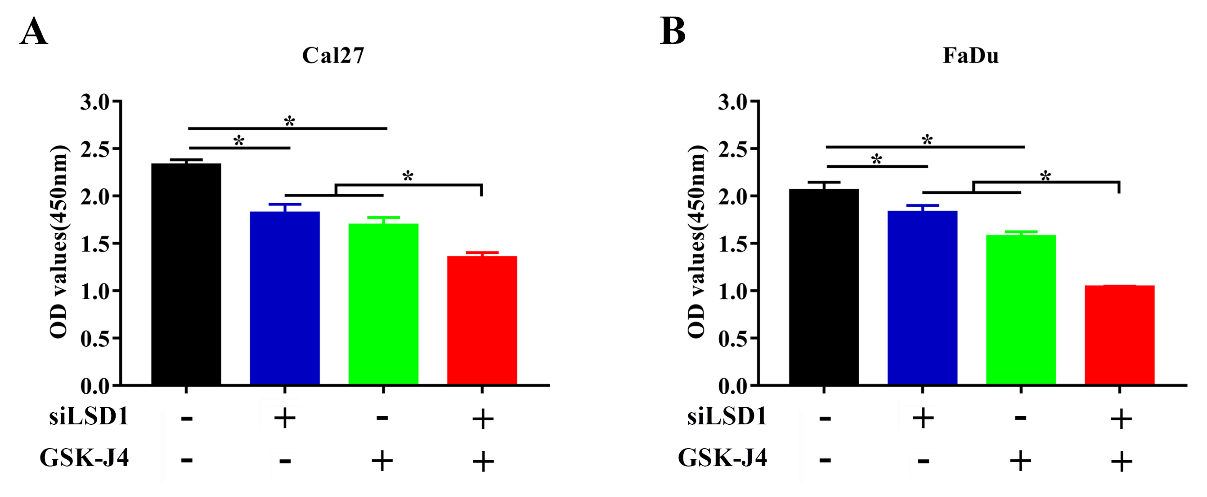


Supplementary Figure 7


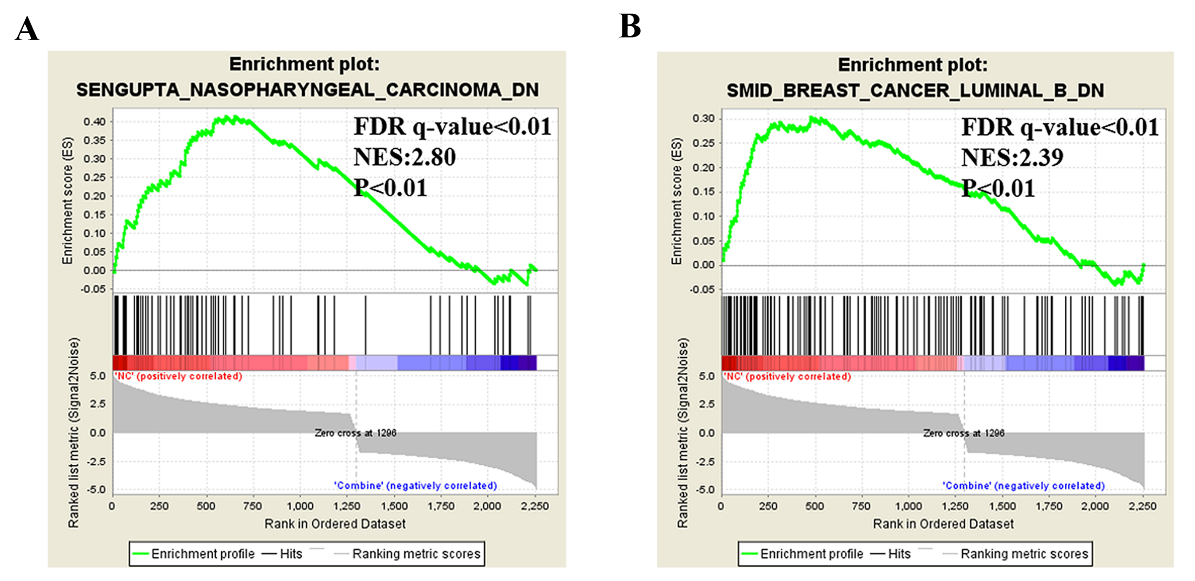


Supplementary Figure 8


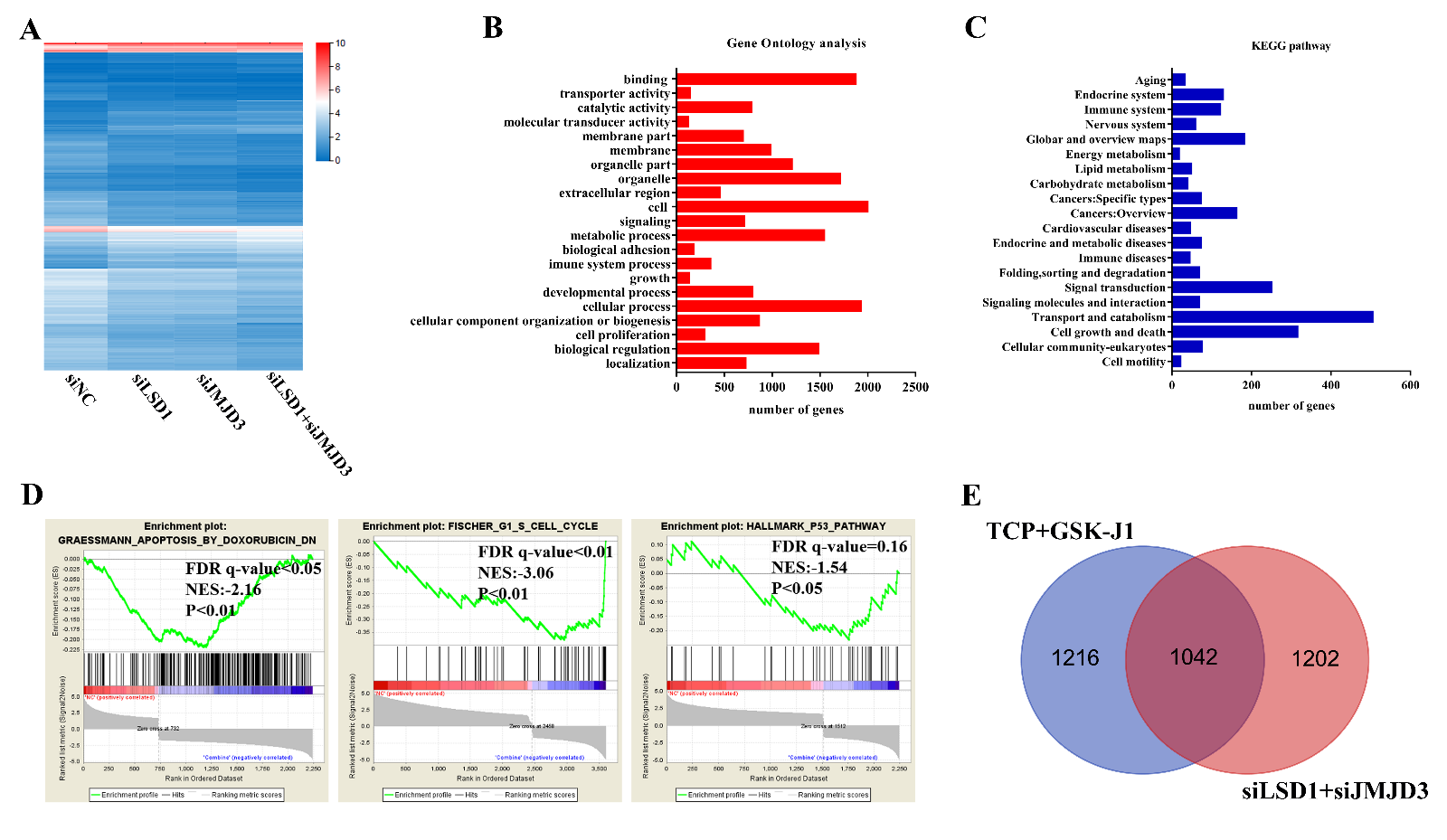


Supplementary Figure 9


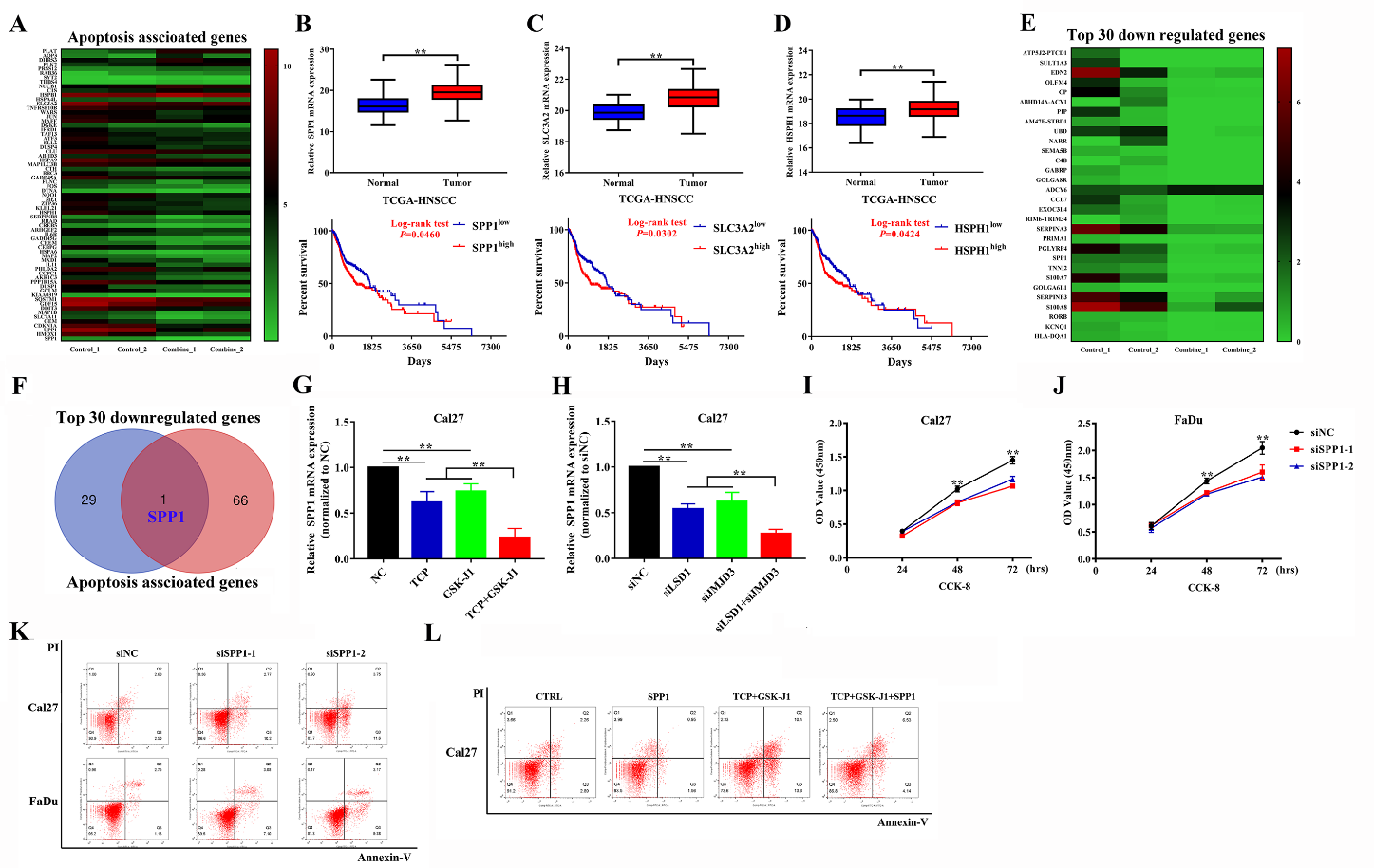


Supplementary Figure 10


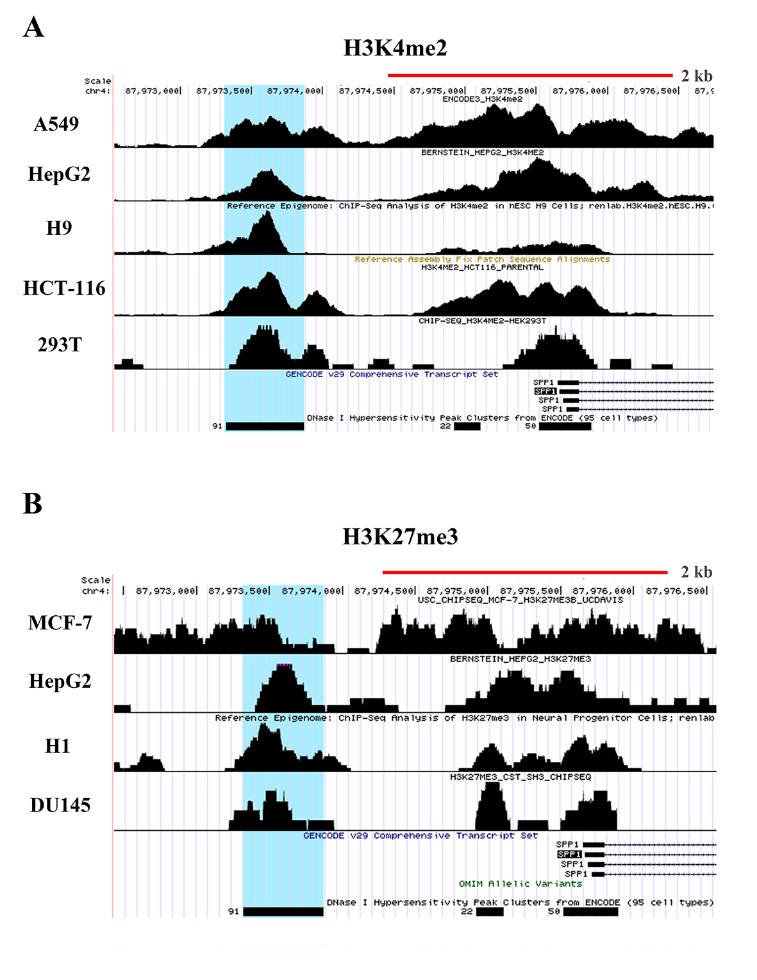


Supplementary Figure 11


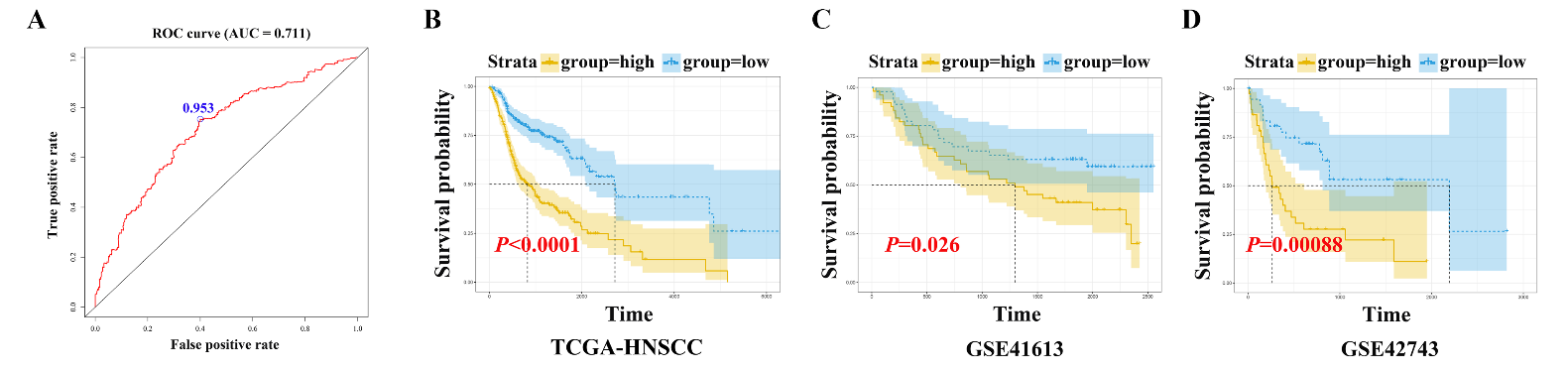


Supplementary Figure 12


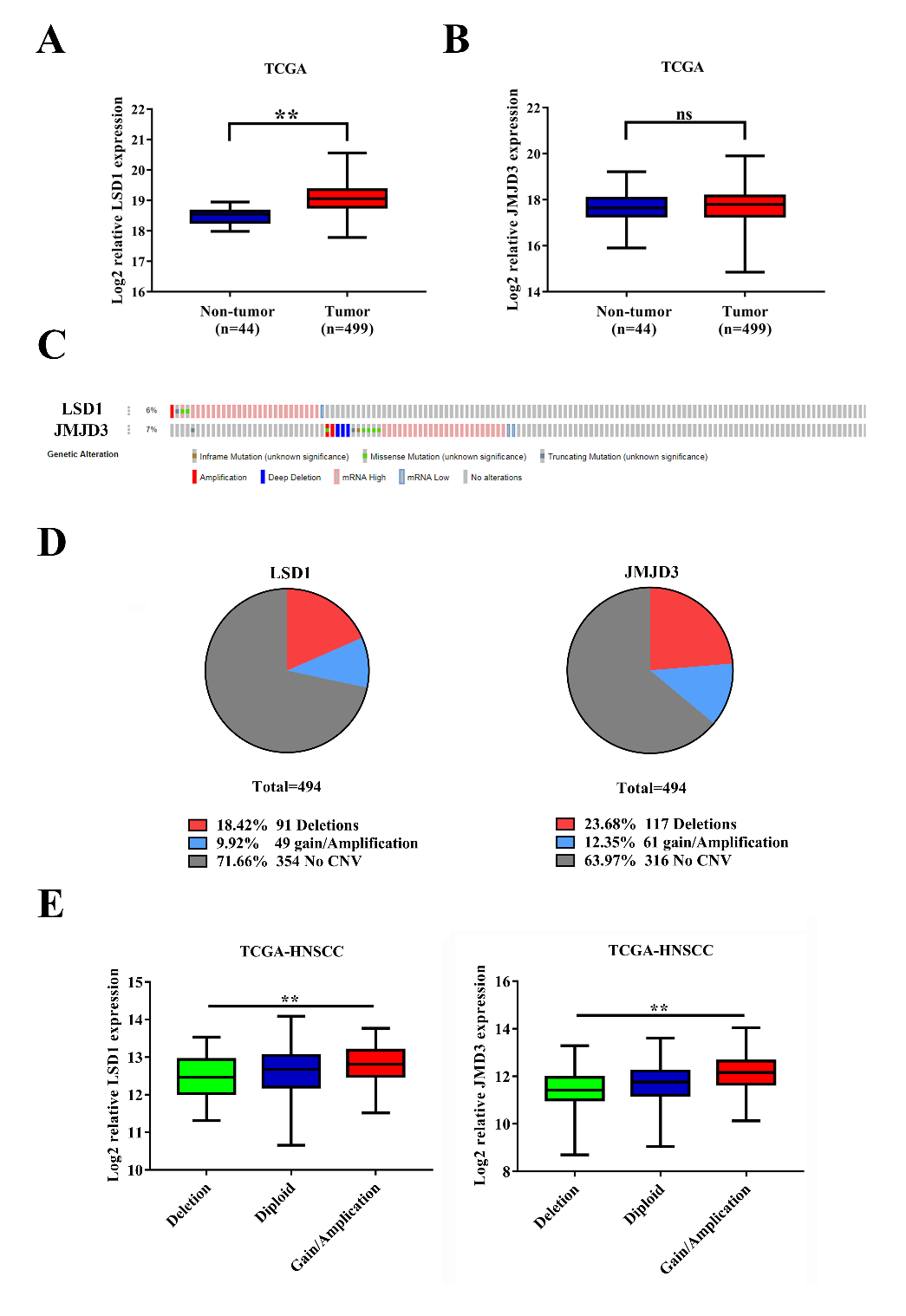


Supplementary Figure 13


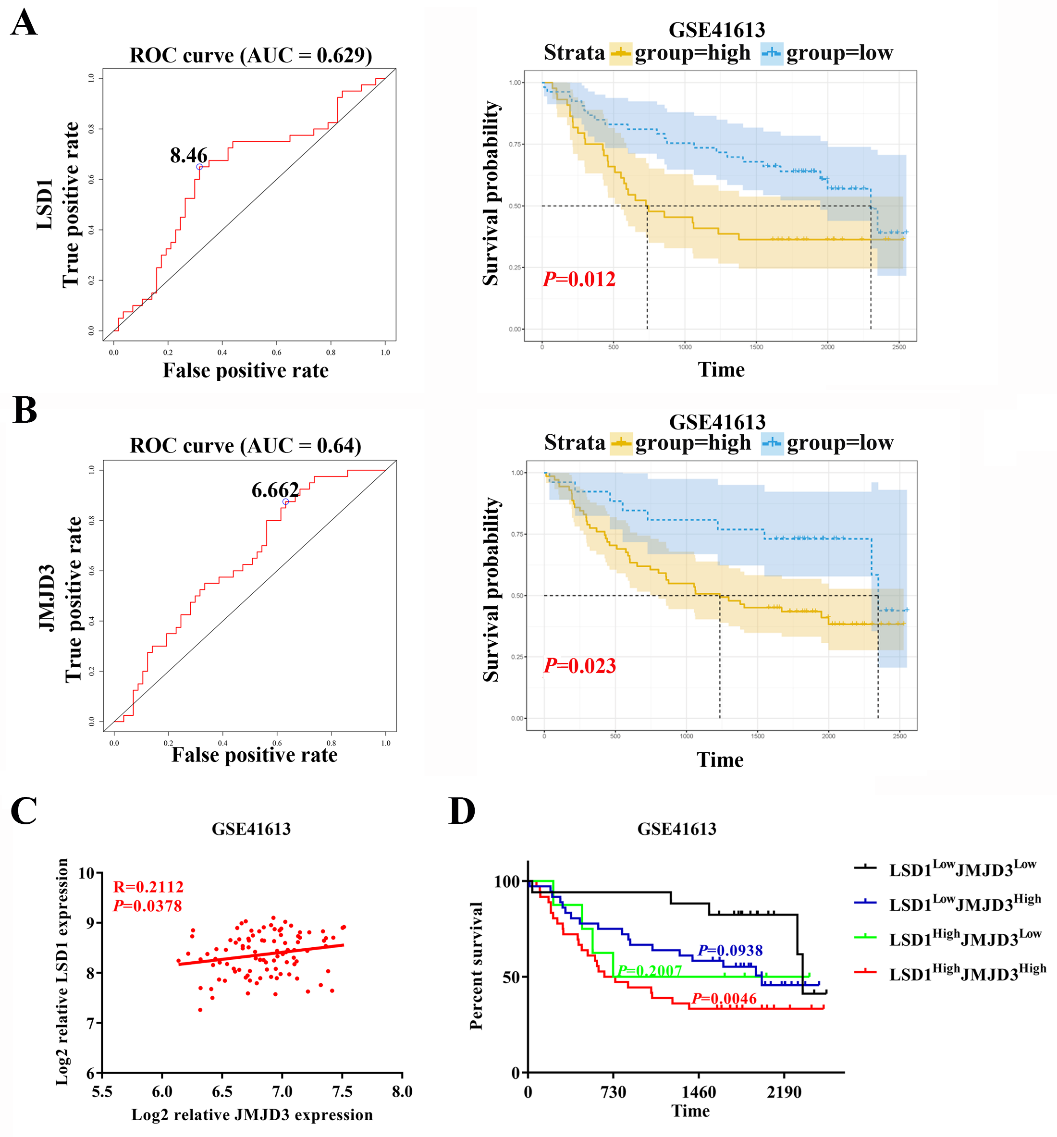


**Supplementary Table Legends**

Supplementary Table 1. IC50 values of these epigenetic compounds in FaDu cells.

Supplementary Table 2. siRNA sequences.

Supplementary Table 3. Antibodies used.

Supplementary Table 4. The clinical and demographic characteristics of TCGA in HNSCC.

Supplementary Table 5. The clinical and demographic characteristics of GSE41613 in HNSCC.

Supplementary Table 6. The clinical and demographic characteristics of GSE42743 in HNSCC.

Supplementary Table 7. Univariate and multivariate survival analyses (proportional hazards method) of LSD1 and JMJD3 expression in prognostic prediction for patients from GSE41613.

**Supplementary Table 1. IC50 values of these epigenetic compounds in FaDu cells**

|  | **Epigenetic compounds** | **Target** | **Compound Supplier** | **Cat. No** | **Average IC50** |
| --- | --- | --- | --- | --- | --- |
| FaDu | Tranylcypromine | KDM1A | Selleckchem | S424605 | 1.2mM |
|  | Decitabine | DNMT1/DNMT3A/DNMT3B | MedChemExpress | HY-A0004 | 442.43nM |
|  | Azacitidine | DNMT1 | MedChemExpress | HY-10586 | 15.06μM |
|  | Panobinostat | HDAC | MedChemExpress | HY-10224 | 59.88nM |
|  | Trichostatin A | HDAC | MedChemExpress | HY-15144 | 118.63nM |
|  | GSK-126 | EZH2 | MedChemExpress | HY-13470 | 51.99μM |
|  | PTC209 | Bmi1 | MedChemExpress | HY-15888 | 5.86μM |
|  | GSK-J1 | KDM6B | MedChemExpress | HY-15648 | 15.05μM |
|  | GSK-J4 | KDM6A/KDM6B | MedChemExpress | HY-15648B | 5.82μM |
|  | JQ1 | BRD4 | MedChemExpress | HY-78695 | 2.85μM |

**Supplementary Table 2. siRNA sequences**

| **siRNA** | **Target** | **Sequence** |
| --- | --- | --- |
| siLSD1-1 | Human LSD1 | ACGAGUCAAACCUUUAUUUTT |
| siLSD1-2 | Human LSD1 | GAAGGCUCUUCUAGCAAUATT |
| siJMJD3-1 | Human JMJD3 | UCCCACUCACCUCUAUUUATT |
| siJMJD3-2 | Human JMJD3 | UGUACAGACCCUCGAAAUCTT |
| siSPP1-1 | Human SPP1 | CGACUCUGAUGAUGUAGAUTT |
| siSPP1-2 | Human SPP1 | GCAUCUUCUGAGGUCAAUUTT |
| Negative control |  | UUCUCCGAACGUGUCACGUTT |

**Supplementary Table 3. Antibodies used.**

| **Specificity** | **Source** | **Catalog number** | **Application** |
| --- | --- | --- | --- |
| Bax | Proteintech | 50599-2-lg | WB (1:2000) |
| Bcl2 | Proteintech | 12789-1-AP | WB (1:1000) |
| Cleaved Caspase3 | Cell signaling | #9661 | WB (1:1000), IHC (1:200) |
| Cleaved PARP | Cell signaling | #5625 | WB (1:1000) |
| p16 | Proteintech | 10883-1-AP | WB (1:1000) |
| p21 | Proteintech | 10355-1-AP | WB (1:1000) |
| Cyclin D1 | Santa Cruz | sc-753 | WB (1:500) |
| LSD1 | Cell signaling | #2184 | WB (1:1000) |
| JMJD3 | Proteintech | 55354-1-AP | WB (1:1000) |
| SPP1 | Proteintech | 25715-1-AP | WB (1:1000), IHC (1:100) |
| H3K27me3 | Cell signaling | #9733 | WB (1:1000), ChIP(1:50) |
| H3K4me2 | Cell signaling | #9725 | WB (1:1000), ChIP(1:50) |
| H3 | Cell signaling | #4499 | WB (1:2000) |
| Ki67 | Dako | M724029-2 | IHC (1:250) |
| β-actin | Santa Cruz | sc-47778 | WB (1:200) |
|  |  |  |  |

**Supplementary Table 4.** **The clinical and demographic characteristics of TCGA in HNSCC**

| Group | Sample Title | Outcome | Time | Gender | Age | Pathologic_stage | Clinical_stage |
| --- | --- | --- | --- | --- | --- | --- | --- |
| Tumor | TCGA-4P-AA8J-01 | 0 | 102 | male | 66 | stage iva | stage iva |
| Tumor | TCGA-BA-5149-01 | 1 | 806 | male | 47 | stage iva | stage iva |
| Tumor | TCGA-BA-5555-01 | 0 | 520 | male | 54 | stage iva | stage iva |
| Tumor | TCGA-BA-5556-01 | 0 | 725 | female | 58 | stage ii | stage ii |
| Tumor | TCGA-BA-5557-01 | 0 | 623 | female | 41 | stage iii | stage iva |
| Tumor | TCGA-BA-6873-01 | 0 | 122 | male | 28 | stage iva | stage iva |
| Tumor | TCGA-BA-7269-01 | 0 | 1273 | male | 61 | stage iii | stage iii |
| Tumor | TCGA-BA-A4II-01 | 0 | 918 | male | 46 | stage ii | stage i |
| Tumor | TCGA-BA-A6D8-01 | 0 | 850 | male | 59 | stage iva | stage iva |
| Tumor | TCGA-BA-A6DA-01 | 0 | 351 | female | 41 | stage iva | stage iva |
| Tumor | TCGA-BA-A6DB-01 | 0 | 216 | female | 24 | stage i | stage ii |
| Tumor | TCGA-BA-A6DD-01 | 1 | 173 | male | 44 | stage iva | stage iva |
| Tumor | TCGA-BA-A6DJ-01 | 1 | 407 | male | 62 | stage iva | stage iva |
| Tumor | TCGA-BB-4228-01 | 0 | 559 | male | 50 | stage iii | stage ii |
| Tumor | TCGA-BB-7871-01 | 0 | 750 | female | 64 | stage iva | stage iva |
| Tumor | TCGA-BB-7872-01 | 0 | 1168 | male | 63 | stage i | stage iva |
| Tumor | TCGA-BB-8601-01 | 0 | 624 | male | 84 | stage iii | stage ii |
| Tumor | TCGA-BB-A5HY-01 | 1 | 321 | male | 64 | stage iva | stage iva |
| Tumor | TCGA-BB-A5HZ-01 | 0 | 827 | male | 65 | stage iva | stage iva |
| Tumor | TCGA-BB-A6UO-01 | 1 | 268 | female | 61 | stage iva | stage iva |
| Tumor | TCGA-C9-A47Z-01 | 1 | 191 | female | 72 | stage iii | stage iii |
| Tumor | TCGA-CN-4722-01 | 0 | 1483 | female | 61 | stage ii | stage ii |
| Tumor | TCGA-CN-4723-01 | 0 | 1699 | male | 67 | stage iva | stage iva |
| Tumor | TCGA-CN-4725-01 | 0 | 1157 | male | 60 | stage ii | stage ii |
| Tumor | TCGA-CN-4728-01 | 0 | 1724 | male | 56 | stage iva | stage iva |
| Tumor | TCGA-CN-4729-01 | 0 | 392 | male | 73 | stage iii | stage iva |
| Tumor | TCGA-CN-4730-01 | 0 | 817 | male | 62 | stage iva | stage iva |
| Tumor | TCGA-CN-4734-01 | 0 | 1690 | male | 70 | stage ii | stage ii |
| Tumor | TCGA-CN-4737-01 | 0 | 625 | male | 19 | stage iva | stage ii |
| Tumor | TCGA-CN-5355-01 | 0 | 1278 | male | 64 | stage iva | stage iva |
| Tumor | TCGA-CN-5363-01 | 1 | 253 | male | 48 | stage ivb | stage ivb |
| Tumor | TCGA-CN-5364-01 | 1 | 493 | male | 55 | stage iva | stage iva |
| Tumor | TCGA-CN-5365-01 | 1 | 351 | male | 38 | stage ivb | stage ivc |
| Tumor | TCGA-CN-5366-01 | 1 | 360 | male | 51 | stage iva | stage iva |
| Tumor | TCGA-CN-5367-01 | 1 | 352 | female | 60 | stage iva | stage iva |
| Tumor | TCGA-CN-5373-01 | 0 | 1584 | female | 55 | stage i | stage ii |
| Tumor | TCGA-CN-5374-01 | 1 | 1732 | female | 56 | stage iva | stage iva |
| Tumor | TCGA-CN-6011-01 | 0 | 933 | male | 57 | stage iva | stage iva |
| Tumor | TCGA-CN-6012-01 | 0 | 1460 | male | 66 | stage iva | stage iii |
| Tumor | TCGA-CN-6018-01 | 1 | 580 | female | 85 | stage iva | stage iva |
| Tumor | TCGA-CN-6020-01 | 1 | 205 | male | 58 | stage iii | stage iva |
| Tumor | TCGA-CN-6024-01 | 1 | 337 | male | 66 | stage iva | stage iva |
| Tumor | TCGA-CN-6995-01 | 1 | 112 | male | 78 | stage iva | stage iva |
| Tumor | TCGA-CN-6996-01 | 1 | 530 | female | 58 | stage iva | stage iva |
| Tumor | TCGA-CN-6998-01 | 1 | 357 | male | 53 | stage iva | stage iva |
| Tumor | TCGA-CN-A498-01 | 1 | 773 | female | 61 | stage iii | stage ii |
| Tumor | TCGA-CN-A63W-01 | 1 | 377 | female | 48 | stage iva | stage iva |
| Tumor | TCGA-CN-A6UY-01 | 0 | 713 | male | 57 | stage iva | stage iva |
| Tumor | TCGA-CN-A6V1-01 | 0 | 603 | male | 59 | stage iva | stage iva |
| Tumor | TCGA-CN-A6V7-01 | 0 | 594 | male | 40 | stage iva | stage iva |
| Tumor | TCGA-CQ-5323-01 | 0 | 1466 | male | 82 | stage i | stage iii |
| Tumor | TCGA-CQ-5324-01 | 0 | 1593 | male | 59 | stage iii | stage iva |
| Tumor | TCGA-CQ-5326-01 | 1 | 89 | male | 67 | stage iva | stage iva |
| Tumor | TCGA-CQ-5327-01 | 0 | 1660 | female | 61 | stage iva | stage iva |
| Tumor | TCGA-CQ-5330-01 | 0 | 1897 | female | 69 | stage iva | stage iii |
| Tumor | TCGA-CQ-5333-01 | 1 | 341 | male | 74 | stage ii | stage i |
| Tumor | TCGA-CQ-5334-01 | 1 | 129 | male | 87 | stage iva | stage ivb |
| Tumor | TCGA-CQ-6218-01 | 0 | 1253 | female | 52 | stage iva | stage iii |
| Tumor | TCGA-CQ-6220-01 | 1 | 985 | male | 69 | stage iii | stage iii |
| Tumor | TCGA-CQ-6222-01 | 0 | 2016 | male | 63 | stage iva | stage iva |
| Tumor | TCGA-CQ-6223-01 | 0 | 1428 | male | 69 | stage ii | stage iva |
| Tumor | TCGA-CQ-6225-01 | 1 | 403 | male | 65 | stage iii | stage ii |
| Tumor | TCGA-CQ-7067-01 | 0 | 509 | female | 75 | stage i | stage i |
| Tumor | TCGA-CQ-7068-01 | 0 | 1309 | female | 80 | stage ii | stage ii |
| Tumor | TCGA-CQ-A4C6-01 | 0 | 1353 | male | 63 | stage ii | stage ii |
| Tumor | TCGA-CQ-A4C7-01 | 1 | 353 | male | 88 | stage iii | stage ii |
| Tumor | TCGA-CQ-A4CB-01 | 0 | 893 | male | 59 | stage iii | stage ii |
| Tumor | TCGA-CQ-A4CD-01 | 0 | 1022 | male | 69 | stage iva | stage ivb |
| Tumor | TCGA-CQ-A4CE-01 | 0 | 897 | female | 76 | stage ii | stage ii |
| Tumor | TCGA-CR-6474-01 | 1 | 564 | male | 51 | stage iva | stage iva |
| Tumor | TCGA-CR-6491-01 | 0 | 693 | male | 60 | stage iva | stage iva |
| Tumor | TCGA-CR-6492-01 | 0 | 479 | male | 78 | stage iii | stage iva |
| Tumor | TCGA-CR-7365-01 | 0 | 1191 | male | 60 | stage iva | stage iva |
| Tumor | TCGA-CR-7369-01 | 1 | 1090 | male | 59 | stage iva | stage iva |
| Tumor | TCGA-CR-7377-01 | 1 | 279 | male | 58 | stage iva | stage iva |
| Tumor | TCGA-CR-7380-01 | 1 | 606 | male | 58 | stage ivb | stage iii |
| Tumor | TCGA-CR-7382-01 | 0 | 796 | male | 49 | stage iva | stage iva |
| Tumor | TCGA-CR-7383-01 | 1 | 521 | female | 79 | stage i | stage i |
| Tumor | TCGA-CR-7390-01 | 0 | 1508 | male | 67 | stage iva | stage iii |
| Tumor | TCGA-CR-7391-01 | 0 | 913 | female | 36 | stage i | stage i |
| Tumor | TCGA-CR-7395-01 | 0 | 930 | female | 80 | stage ii | stage ii |
| Tumor | TCGA-CV-5432-01 | 0 | 3930 | male | 68 | stage iii | stage iii |
| Tumor | TCGA-CV-5435-01 | 1 | 2319 | male | 57 | stage iva | stage iva |
| Tumor | TCGA-CV-5436-01 | 1 | 584 | male | 65 | stage iva | stage iva |
| Tumor | TCGA-CV-5439-01 | 1 | 546 | male | 62 | stage iva | stage ii |
| Tumor | TCGA-CV-5442-01 | 0 | 2327 | female | 76 | stage iva | stage iva |
| Tumor | TCGA-CV-5443-01 | 0 | 2784 | male | 63 | stage iii | stage iii |
| Tumor | TCGA-CV-5444-01 | 0 | 2437 | male | 64 | stage iva | stage iva |
| Tumor | TCGA-CV-5966-01 | 1 | 545 | female | 63 | stage iva | stage iva |
| Tumor | TCGA-CV-5970-01 | 1 | 406 | male | 59 | stage iva | stage iva |
| Tumor | TCGA-CV-5973-01 | 0 | 2641 | female | 62 | stage iii | stage iii |
| Tumor | TCGA-CV-5976-01 | 0 | 1478 | male | 50 | stage iva | stage iii |
| Tumor | TCGA-CV-5977-01 | 0 | 1840 | male | 66 | stage iva | stage iii |
| Tumor | TCGA-CV-5979-01 | 0 | 1315 | male | 26 | stage iva | stage iii |
| Tumor | TCGA-CV-6436-01 | 0 | 1899 | male | 62 | stage iva | stage iii |
| Tumor | TCGA-CV-6933-01 | 1 | 2741 | male | 53 | stage iii | stage iva |
| Tumor | TCGA-CV-6935-01 | 1 | 295 | male | 67 | stage iva | stage iii |
| Tumor | TCGA-CV-6936-01 | 1 | 166 | male | 68 | stage iva | stage iva |
| Tumor | TCGA-CV-6938-01 | 1 | 144 | male | 87 | stage ii | stage ii |
| Tumor | TCGA-CV-6940-01 | 1 | 804 | female | 80 | stage iii | stage i |
| Tumor | TCGA-CV-6941-01 | 1 | 342 | male | 51 | stage iii | stage iii |
| Tumor | TCGA-CV-6945-01 | 1 | 366 | male | 41 | stage iva | stage iva |
| Tumor | TCGA-CV-6950-01 | 1 | 459 | male | 64 | stage iva | stage iva |
| Tumor | TCGA-CV-6952-01 | 1 | 185 | female | 65 | stage iva | stage iii |
| Tumor | TCGA-CV-6953-01 | 1 | 1641 | female | 80 | stage iii | stage iii |
| Tumor | TCGA-CV-6955-01 | 1 | 334 | female | 74 | stage ii | stage ii |
| Tumor | TCGA-CV-6960-01 | 1 | 862 | male | 49 | stage iii | stage iii |
| Tumor | TCGA-CV-7089-01 | 1 | 1972 | male | 74 | stage iva | stage iva |
| Tumor | TCGA-CV-7090-01 | 0 | 5252 | male | 39 | stage ii | stage ii |
| Tumor | TCGA-CV-7095-01 | 1 | 572 | female | 87 | stage ii | stage iva |
| Tumor | TCGA-CV-7097-01 | 1 | 385 | male | 53 | stage ii | stage iii |
| Tumor | TCGA-CV-7099-01 | 1 | 243 | female | 85 | stage ii | stage ii |
| Tumor | TCGA-CV-7100-01 | 1 | 274 | male | 66 | stage iii | stage ii |
| Tumor | TCGA-CV-7102-01 | 1 | 56 | female | 76 | stage iva | stage iii |
| Tumor | TCGA-CV-7104-01 | 1 | 393 | female | 61 | stage iva | stage iva |
| Tumor | TCGA-CV-7177-01 | 1 | 663 | female | 82 | stage ii | stage i |
| Tumor | TCGA-CV-7178-01 | 1 | 2166 | female | 64 | stage iva | stage iva |
| Tumor | TCGA-CV-7180-01 | 1 | 327 | male | 34 | stage ii | stage ii |
| Tumor | TCGA-CV-7183-01 | 0 | 3981 | male | 53 | stage ii | stage ii |
| Tumor | TCGA-CV-7245-01 | 0 | 797 | male | 62 | stage iva | stage iii |
| Tumor | TCGA-CV-7247-01 | 1 | 577 | male | 55 | stage iii | stage ii |
| Tumor | TCGA-CV-7263-01 | 1 | 560 | male | 64 | stage ii | stage ii |
| Tumor | TCGA-CV-7407-01 | 1 | 1081 | female | 67 | stage ii | stage ii |
| Tumor | TCGA-CV-7411-01 | 1 | 2717 | female | 64 | stage iva | stage iva |
| Tumor | TCGA-CV-7418-01 | 1 | 789 | male | 62 | stage iva | stage iva |
| Tumor | TCGA-CV-7423-01 | 1 | 3059 | male | 65 | stage ii | stage ii |
| Tumor | TCGA-CV-7425-01 | 1 | 1718 | female | 77 | stage iii | stage iii |
| Tumor | TCGA-CV-7427-01 | 1 | 4760 | female | 73 | stage ii | stage ii |
| Tumor | TCGA-CV-7428-01 | 1 | 1671 | male | 47 | stage iva | stage iva |
| Tumor | TCGA-CV-7432-01 | 1 | 2570 | male | 79 | stage ii | stage iii |
| Tumor | TCGA-CV-7434-01 | 1 | 218 | male | 64 | stage iva | stage iii |
| Tumor | TCGA-CV-7438-01 | 1 | 194 | female | 87 | stage i | stage ii |
| Tumor | TCGA-CV-A45P-01 | 0 | 639 | female | 82 | stage i | stage ii |
| Tumor | TCGA-CV-A45T-01 | 1 | 4856 | female | 64 | stage ii | stage ii |
| Tumor | TCGA-CV-A45V-01 | 1 | 32 | female | 87 | stage iva | stage iva |
| Tumor | TCGA-CV-A45W-01 | 1 | 1398 | male | 75 | stage ii | stage iii |
| Tumor | TCGA-CV-A45X-01 | 1 | 198 | male | 47 | stage iva | stage iva |
| Tumor | TCGA-CV-A45Y-01 | 1 | 2703 | male | 61 | stage iva | stage iva |
| Tumor | TCGA-CV-A463-01 | 1 | 23 | female | 82 | stage iva | stage iva |
| Tumor | TCGA-CV-A465-01 | 1 | 215 | male | 24 | stage iii | stage ii |
| Tumor | TCGA-CV-A468-01 | 1 | 464 | male | 42 | stage iva | stage iii |
| Tumor | TCGA-CV-A6JO-01 | 1 | 197 | male | 69 | stage iva | stage iva |
| Tumor | TCGA-CV-A6JT-01 | 0 | 852 | male | 65 | stage ii | stage ii |
| Tumor | TCGA-CV-A6JY-01 | 0 | 646 | male | 69 | stage iva | stage iva |
| Tumor | TCGA-CV-A6JZ-01 | 0 | 714 | male | 68 | stage ii | stage iva |
| Tumor | TCGA-CV-A6K1-01 | 0 | 685 | male | 65 | stage iva | stage iva |
| Tumor | TCGA-CX-7219-01 | 0 | 1045 | male | 47 | stage iva | stage iva |
| Tumor | TCGA-D6-6516-01 | 0 | 773 | male | 69 | stage i | stage iii |
| Tumor | TCGA-D6-6517-01 | 0 | 292 | male | 59 | stage iii | stage iii |
| Tumor | TCGA-D6-6823-01 | 0 | 701 | male | 50 | stage ii | stage iii |
| Tumor | TCGA-D6-6825-01 | 0 | 491 | male | 73 | stage i | stage iii |
| Tumor | TCGA-D6-6826-01 | 1 | 348 | female | 64 | stage iva | stage iva |
| Tumor | TCGA-D6-6827-01 | 0 | 568 | female | 55 | stage i | stage iii |
| Tumor | TCGA-D6-8568-01 | 0 | 759 | male | 62 | stage ii | stage ii |
| Tumor | TCGA-D6-A4Z9-01 | 0 | 539 | male | 59 | stage iva | stage iva |
| Tumor | TCGA-D6-A4ZB-01 | 0 | 376 | male | 61 | stage iii | stage iii |
| Tumor | TCGA-D6-A6EK-01 | 0 | 875 | male | 67 | stage iii | stage iva |
| Tumor | TCGA-D6-A6EM-01 | 0 | 232 | female | 65 | stage iii | stage iii |
| Tumor | TCGA-D6-A6EN-01 | 0 | 687 | male | 71 | stage iii | stage iii |
| Tumor | TCGA-D6-A6EQ-01 | 0 | 368 | male | 57 | stage iva | stage iva |
| Tumor | TCGA-D6-A6ES-01 | 0 | 389 | male | 50 | stage iva | stage iva |
| Tumor | TCGA-F7-8489-01 | 0 | 658 | male | 48 | stage ii | stage ii |
| Tumor | TCGA-H7-7774-01 | 0 | 407 | female | 75 | stage iva | stage iii |
| Tumor | TCGA-H7-8502-01 | 0 | 458 | male | 50 | stage iva | stage iva |
| Tumor | TCGA-H7-A6C4-01 | 1 | NA | female | 35 | stage iva | stage iii |
| Tumor | TCGA-HD-7229-01 | 0 | 1027 | male | 60 | stage iva | stage iva |
| Tumor | TCGA-HD-7832-01 | 0 | 836 | male | 52 | stage iva | stage iva |
| Tumor | TCGA-HD-8314-01 | 0 | 670 | male | 58 | stage iii | stage iii |
| Tumor | TCGA-HD-8634-01 | 1 | 385 | female | 51 | stage i | stage i |
| Tumor | TCGA-HD-8635-01 | 0 | 695 | female | 61 | stage iii | stage iii |
| Tumor | TCGA-HD-A4C1-01 | 0 | 11 | female | 41 | stage iva | stage iva |
| Tumor | TCGA-HD-A633-01 | 0 | 421 | male | 74 | stage iva | stage iva |
| Tumor | TCGA-HD-A6I0-01 | 0 | 210 | male | 56 | stage iva | stage iii |
| Tumor | TCGA-IQ-7631-01 | 0 | 1172 | female | 60 | stage ii | stage ii |
| Tumor | TCGA-IQ-A61I-01 | 1 | 2 | male | 63 | stage iva | stage iva |
| Tumor | TCGA-IQ-A61J-01 | 0 | 1021 | male | 54 | stage iva | stage iva |
| Tumor | TCGA-IQ-A6SG-01 | 0 | 579 | female | 61 | stage iii | stage iii |
| Tumor | TCGA-IQ-A6SH-01 | 0 | 471 | male | 55 | stage iii | stage ivc |
| Tumor | TCGA-KU-A6H7-01 | 0 | 586 | female | 55 | stage iva | stage iva |
| Tumor | TCGA-MT-A51W-01 | 0 | 437 | female | 52 | stage i | stage i |
| Tumor | TCGA-MT-A51X-01 | 0 | 242 | male | 30 | stage iva | stage iva |
| Tumor | TCGA-MT-A67A-01 | 0 | 914 | female | 85 | stage i | stage i |
| Tumor | TCGA-MT-A67D-01 | 0 | 56 | male | 55 | stage ii | stage ii |
| Tumor | TCGA-MT-A67F-01 | 0 | 384 | female | 60 | stage iva | stage iva |
| Tumor | TCGA-P3-A5Q6-01 | 1 | 480 | male | 49 | stage iva | stage iii |
| Tumor | TCGA-P3-A5QA-01 | 0 | 2182 | male | 41 | stage i | stage ii |
| Tumor | TCGA-P3-A6SW-01 | 0 | 1120 | male | 50 | stage ivb | stage iva |
| Tumor | TCGA-P3-A6SX-01 | 1 | 1430 | male | 67 | stage iii | stage iva |
| Tumor | TCGA-P3-A6T2-01 | 0 | 2298 | male | 45 | stage iva | stage ii |
| Tumor | TCGA-P3-A6T3-01 | 1 | 577 | male | 49 | stage iva | stage iva |
| Tumor | TCGA-P3-A6T5-01 | 1 | 882 | female | 79 | stage iva | stage iva |
| Tumor | TCGA-P3-A6T7-01 | 1 | 487 | male | 55 | stage iva | stage iii |
| Tumor | TCGA-QK-A64Z-01 | 1 | 641 | female | 79 | stage iva | stage ii |
| Tumor | TCGA-QK-A652-01 | 0 | 645 | male | 60 | stage iii | stage ii |
| Tumor | TCGA-QK-A6IH-01 | 0 | 653 | female | 65 | stage ivb | stage iva |
| Tumor | TCGA-QK-A6II-01 | 1 | 284 | male | 52 | stage iva | stage iii |
| Tumor | TCGA-QK-A6IJ-01 | 0 | 387 | male | 71 | stage iii | stage ii |
| Tumor | TCGA-QK-A6V9-01 | 0 | 833 | male | 56 | stage ii | stage ii |
| Tumor | TCGA-QK-A8ZB-01 | 0 | 542 | male | 68 | stage iva | stage iva |
| Tumor | TCGA-QK-AA3K-01 | 0 | 253 | male | 60 | stage iva | stage iva |
| Tumor | TCGA-RS-A6TO-01 | 1 | 387 | female | 82 | stage iva | stage iva |
| Tumor | TCGA-T2-A6X2-01 | 0 | 987 | male | 82 | stage iii | stage iii |
| Tumor | TCGA-T3-A92N-01 | 1 | 95 | male | 79 | stage iva | stage ivc |
| Tumor | TCGA-TN-A7HI-01 | 0 | 412 | male | 56 | stage iva | stage i |
| Tumor | TCGA-TN-A7HJ-01 | 0 | 403 | male | 51 | stage iva | stage iii |
| Tumor | TCGA-TN-A7HL-01 | 0 | 619 | male | 59 | stage iva | stage iva |
| Tumor | TCGA-UF-A718-01 | 0 | 1971 | male | 62 | stage iva | stage iva |
| Tumor | TCGA-UF-A71A-01 | 1 | 86 | male | 67 | stage iva | stage iva |
| Tumor | TCGA-UF-A71B-01 | 0 | 1506 | male | 50 | stage iva | stage iva |
| Tumor | TCGA-UF-A71E-01 | 1 | 1504 | male | 63 | stage iva | stage iva |
| Tumor | TCGA-UF-A7JC-01 | 1 | 546 | male | 42 | stage iva | stage iii |
| Tumor | TCGA-UF-A7JF-01 | 0 | 1686 | male | 80 | stage iva | stage iva |
| Tumor | TCGA-UF-A7JH-01 | 0 | 896 | male | 59 | stage iva | stage iva |
| Tumor | TCGA-UF-A7JO-01 | 1 | 631 | female | 79 | stage iva | stage iva |
| Tumor | TCGA-UF-A7JS-01 | 1 | 680 | male | 59 | stage iva | stage iva |
| Tumor | TCGA-UF-A7JT-01 | 1 | 993 | female | 72 | stage iva | stage iva |
| Tumor | TCGA-UF-A7JV-01 | 1 | 90 | female | 62 | stage iva | stage iva |
| Tumor | TCGA-BA-4074-01 | 1 | 462 | male | 69 | stage iva | stage iva |
| Tumor | TCGA-BA-4075-01 | 1 | 283 | male | 49 | stage iii | stage iva |
| Tumor | TCGA-BA-4077-01 | 1 | 1134 | female | 45 | stage iva | stage ivb |
| Tumor | TCGA-BA-5151-01 | 0 | 722 | male | 72 | stage iva | stage iva |
| Tumor | TCGA-BA-5152-01 | 0 | 1288 | male | 56 | stage iva | stage iva |
| Tumor | TCGA-BA-6869-01 | 0 | 644 | male | 62 | stage iii | stage iii |
| Tumor | TCGA-BA-A6DE-01 | 0 | 440 | female | 70 | stage ii | stage iii |
| Tumor | TCGA-BA-A8YP-01 | 0 | 499 | male | 50 | stage ivb | stage ivb |
| Tumor | TCGA-BB-4217-01 | 0 | 187 | male | 68 | stage iva | stage iva |
| Tumor | TCGA-BB-4223-01 | 0 | 3221 | male | 48 | stage iva | stage iva |
| Tumor | TCGA-BB-4224-01 | 0 | 278 | male | 52 | stage iva | stage iii |
| Tumor | TCGA-BB-4227-01 | 0 | 134 | male | 66 | stage iva | stage iva |
| Tumor | TCGA-BB-7861-01 | 0 | 682 | male | 56 | stage iii | stage iii |
| Tumor | TCGA-BB-7862-01 | 0 | 1117 | male | 67 | stage iva | stage iva |
| Tumor | TCGA-BB-7863-01 | 0 | 1025 | female | 43 | stage iii | stage iii |
| Tumor | TCGA-BB-7864-01 | 0 | 1527 | male | 61 | stage iva | stage iva |
| Tumor | TCGA-BB-7870-01 | 0 | 2016 | male | 58 | stage iva | stage iva |
| Tumor | TCGA-BB-8596-01 | 0 | 2161 | female | 69 | stage iva | stage iva |
| Tumor | TCGA-BB-A5HU-01 | 0 | 782 | male | 47 | stage iva | stage iva |
| Tumor | TCGA-BB-A6UM-01 | 0 | 393 | male | 52 | stage iii | stage iii |
| Tumor | TCGA-C9-A480-01 | 0 | 386 | female | 45 | stage iii | stage iii |
| Tumor | TCGA-CN-4726-01 | 1 | 142 | male | 68 | stage iva | stage iva |
| Tumor | TCGA-CN-4727-01 | 0 | 1560 | male | 56 | stage iva | stage iva |
| Tumor | TCGA-CN-4731-01 | 1 | 998 | female | 63 | stage iva | stage iva |
| Tumor | TCGA-CN-4733-01 | 0 | 1586 | male | 61 | stage iii | stage i |
| Tumor | TCGA-CN-4735-01 | 0 | 1737 | male | 52 | stage iva | stage iva |
| Tumor | TCGA-CN-4738-01 | 1 | 436 | male | 53 | stage iva | stage iva |
| Tumor | TCGA-CN-4739-01 | 1 | 1394 | male | 71 | stage iva | stage iva |
| Tumor | TCGA-CN-4740-01 | 1 | 839 | female | 79 | stage iva | stage iva |
| Tumor | TCGA-CN-4741-01 | 0 | 2239 | male | 75 | stage iva | stage iva |
| Tumor | TCGA-CN-4742-01 | 1 | 397 | female | 48 | stage iva | stage iva |
| Tumor | TCGA-CN-5356-01 | 0 | 1409 | male | 56 | stage iii | stage iii |
| Tumor | TCGA-CN-5358-01 | 1 | 261 | male | 60 | stage ii | stage iii |
| Tumor | TCGA-CN-5359-01 | 1 | 377 | male | 59 | stage iva | stage iva |
| Tumor | TCGA-CN-5360-01 | 0 | 2169 | male | 68 | stage iva | stage iva |
| Tumor | TCGA-CN-5369-01 | 1 | 380 | female | 90 | stage iva | stage iva |
| Tumor | TCGA-CN-5370-01 | 1 | 259 | male | 78 | stage iii | stage ii |
| Tumor | TCGA-CN-6010-01 | 0 | 1523 | male | 53 | stage iva | stage iva |
| Tumor | TCGA-CN-6013-01 | 1 | 727 | male | 56 | stage iva | stage iva |
| Tumor | TCGA-CN-6016-01 | 0 | 1443 | male | 64 | stage iva | stage iva |
| Tumor | TCGA-CN-6017-01 | 1 | 853 | male | 55 | stage iva | stage iii |
| Tumor | TCGA-CN-6019-01 | 0 | 1038 | male | 61 | stage iva | stage iva |
| Tumor | TCGA-CN-6022-01 | 1 | 281 | male | 49 | stage iva | stage iva |
| Tumor | TCGA-CN-6023-01 | 0 | 1584 | male | 73 | stage iva | stage iva |
| Tumor | TCGA-CN-6988-01 | 0 | 318 | male | 47 | stage iva | stage iva |
| Tumor | TCGA-CN-6989-01 | 1 | 980 | male | 64 | stage iva | stage iva |
| Tumor | TCGA-CN-6992-01 | 0 | 1066 | male | 61 | stage iva | stage iva |
| Tumor | TCGA-CN-6994-01 | 0 | 1183 | male | 67 | stage iva | stage iva |
| Tumor | TCGA-CN-6997-01 | 1 | 988 | male | 66 | stage iva | stage iva |
| Tumor | TCGA-CN-A497-01 | 0 | 1065 | male | 63 | stage iii | stage iva |
| Tumor | TCGA-CN-A499-01 | 0 | 717 | female | 60 | stage ii | stage i |
| Tumor | TCGA-CN-A49A-01 | 1 | 526 | male | 60 | stage ivb | stage iva |
| Tumor | TCGA-CN-A49B-01 | 0 | 904 | male | 71 | stage iii | stage iii |
| Tumor | TCGA-CN-A63T-01 | 0 | 225 | male | 60 | stage iva | stage iva |
| Tumor | TCGA-CN-A63U-01 | 0 | 964 | male | 50 | stage iva | stage iii |
| Tumor | TCGA-CN-A63V-01 | 0 | 679 | male | 59 | stage iva | stage iva |
| Tumor | TCGA-CN-A641-01 | 0 | 367 | male | 47 | stage iva | stage iva |
| Tumor | TCGA-CN-A642-01 | 1 | 82 | male | 57 | stage ivb | stage iva |
| Tumor | TCGA-CN-A6V3-01 | 0 | 742 | male | 61 | stage iva | stage iva |
| Tumor | TCGA-CN-A6V6-01 | 0 | 635 | male | 59 | stage iva | stage iva |
| Tumor | TCGA-CQ-5325-01 | 1 | 654 | male | 65 | stage i | stage i |
| Tumor | TCGA-CQ-5329-01 | 0 | 2143 | female | 46 | stage ii | stage iii |
| Tumor | TCGA-CQ-5332-01 | 1 | 317 | male | 87 | stage iii | stage iii |
| Tumor | TCGA-CQ-6219-01 | 1 | 479 | female | 50 | stage iva | stage iva |
| Tumor | TCGA-CQ-6224-01 | 0 | 1721 | male | 52 | stage iva | stage ii |
| Tumor | TCGA-CQ-6227-01 | 1 | 129 | male | 77 | stage iva | stage iii |
| Tumor | TCGA-CQ-6228-01 | 1 | 456 | female | 71 | stage iva | stage iii |
| Tumor | TCGA-CQ-6229-01 | 0 | 1179 | male | 61 | stage ii | stage iii |
| Tumor | TCGA-CQ-7065-01 | 0 | 1628 | male | 40 | stage ii | stage ii |
| Tumor | TCGA-CQ-7069-01 | 0 | 1274 | female | 77 | stage ii | stage ii |
| Tumor | TCGA-CQ-7071-01 | 0 | 1311 | female | 76 | stage iii | stage iva |
| Tumor | TCGA-CQ-7072-01 | 0 | 2359 | male | 51 | stage ii | stage iva |
| Tumor | TCGA-CQ-A4CG-01 | 1 | 430 | female | 78 | stage iii | stage ii |
| Tumor | TCGA-CQ-A4CH-01 | 1 | 379 | male | 58 | stage ii | stage iva |
| Tumor | TCGA-CR-5249-01 | 0 | 1152 | female | 35 | stage ii | stage ii |
| Tumor | TCGA-CR-6467-01 | 0 | 1777 | male | 59 | stage iii | stage iva |
| Tumor | TCGA-CR-6470-01 | 0 | 1521 | male | 38 | stage ii | stage iva |
| Tumor | TCGA-CR-6471-01 | 1 | 1202 | male | 58 | stage iva | stage iva |
| Tumor | TCGA-CR-6484-01 | 0 | 354 | female | 67 | stage iva | stage iva |
| Tumor | TCGA-CR-6487-01 | 0 | 234 | male | 50 | stage i | stage ii |
| Tumor | TCGA-CR-6488-01 | 0 | 379 | female | 68 | stage ii | stage ii |
| Tumor | TCGA-CR-6493-01 | 1 | 282 | male | 69 | stage iva | stage iva |
| Tumor | TCGA-CR-7364-01 | 0 | 1435 | male | 66 | stage iii | stage iii |
| Tumor | TCGA-CR-7367-01 | 0 | 1440 | male | 52 | stage iva | stage ivb |
| Tumor | TCGA-CR-7368-01 | 0 | 1245 | male | 54 | stage iva | stage iva |
| Tumor | TCGA-CR-7371-01 | 1 | 94 | female | 45 | stage iii | stage iii |
| Tumor | TCGA-CR-7372-01 | 0 | 759 | male | 45 | stage i | stage ii |
| Tumor | TCGA-CR-7373-01 | 0 | 889 | male | 66 | stage iva | stage iva |
| Tumor | TCGA-CR-7376-01 | 0 | 972 | male | 83 | stage iii | stage ii |
| Tumor | TCGA-CR-7379-01 | 0 | 1036 | female | 78 | stage iva | stage iva |
| Tumor | TCGA-CR-7386-01 | 0 | 1430 | male | 69 | stage iva | stage iva |
| Tumor | TCGA-CR-7392-01 | 0 | 1425 | female | 67 | stage iva | stage iva |
| Tumor | TCGA-CR-7393-01 | 0 | 993 | male | 26 | stage iii | stage i |
| Tumor | TCGA-CR-7394-01 | 0 | 1346 | male | 70 | stage iva | stage iva |
| Tumor | TCGA-CR-7397-01 | 0 | 754 | male | 44 | stage iva | stage iva |
| Tumor | TCGA-CR-7398-01 | 0 | 156 | female | 53 | stage ii | stage ii |
| Tumor | TCGA-CR-7399-01 | 0 | 181 | female | 60 | stage iva | stage iva |
| Tumor | TCGA-CR-7401-01 | 0 | 1077 | male | 64 | stage i | stage i |
| Tumor | TCGA-CV-5430-01 | 0 | 4241 | male | 61 | stage iva | stage iva |
| Tumor | TCGA-CV-5431-01 | 1 | 522 | male | 73 | stage iva | stage iva |
| Tumor | TCGA-CV-5434-01 | 1 | 3314 | male | 60 | stage iva | stage iva |
| Tumor | TCGA-CV-5440-01 | 0 | 3270 | male | 52 | stage iva | stage iva |
| Tumor | TCGA-CV-5441-01 | 0 | 2886 | male | 58 | stage iva | stage iva |
| Tumor | TCGA-CV-5971-01 | 0 | 701 | male | 60 | stage iva | stage iva |
| Tumor | TCGA-CV-5978-01 | 1 | 215 | female | 53 | stage ivb | stage ivb |
| Tumor | TCGA-CV-6003-01 | 0 | 1665 | female | 50 | stage iii | stage ii |
| Tumor | TCGA-CV-6433-01 | 0 | 641 | male | 57 | stage ii | stage ii |
| Tumor | TCGA-CV-6441-01 | 1 | 292 | male | 60 | stage iii | stage iii |
| Tumor | TCGA-CV-6934-01 | 1 | 65 | female | 66 | stage iva | stage iva |
| Tumor | TCGA-CV-6937-01 | 1 | 624 | male | 71 | stage ii | stage ii |
| Tumor | TCGA-CV-6939-01 | 1 | 666 | male | 60 | stage iva | stage iva |
| Tumor | TCGA-CV-6942-01 | 0 | 4282 | female | 73 | stage ii | stage ii |
| Tumor | TCGA-CV-6943-01 | 1 | 602 | male | 74 | stage iii | stage iii |
| Tumor | TCGA-CV-6948-01 | 1 | 1289 | female | 79 | stage ivb | stage iva |
| Tumor | TCGA-CV-6951-01 | 1 | 915 | male | 57 | stage iva | stage iva |
| Tumor | TCGA-CV-6954-01 | 1 | 2002 | male | 59 | stage iva | stage iva |
| Tumor | TCGA-CV-6956-01 | 1 | 217 | male | 67 | stage iii | stage iva |
| Tumor | TCGA-CV-6959-01 | 1 | 256 | male | 48 | stage iii | stage iii |
| Tumor | TCGA-CV-6961-01 | 1 | 76 | male | 61 | stage ii | stage ii |
| Tumor | TCGA-CV-6962-01 | 1 | 126 | male | 65 | stage iva | stage iii |
| Tumor | TCGA-CV-7091-01 | 0 | 3381 | male | 54 | stage i | stage i |
| Tumor | TCGA-CV-7101-01 | 1 | 160 | male | 80 | stage ii | stage ii |
| Tumor | TCGA-CV-7103-01 | 1 | 1591 | male | 49 | stage iva | stage ii |
| Tumor | TCGA-CV-7235-01 | 0 | 2347 | male | 67 | stage ii | stage iii |
| Tumor | TCGA-CV-7236-01 | 1 | 144 | female | 77 | stage iva | stage iva |
| Tumor | TCGA-CV-7238-01 | 0 | 2727 | female | 69 | stage ii | stage ii |
| Tumor | TCGA-CV-7242-01 | 0 | 1095 | female | 60 | stage ii | stage iii |
| Tumor | TCGA-CV-7243-01 | 0 | 954 | male | 50 | stage iii | stage ii |
| Tumor | TCGA-CV-7248-01 | 1 | 521 | female | 63 | stage iva | stage iva |
| Tumor | TCGA-CV-7250-01 | 1 | 2900 | male | 64 | stage iva | stage iii |
| Tumor | TCGA-CV-7252-01 | 1 | 151 | female | 62 | stage iva | stage iii |
| Tumor | TCGA-CV-7253-01 | 1 | 361 | male | 58 | stage iva | stage ii |
| Tumor | TCGA-CV-7254-01 | 1 | 1459 | male | 55 | stage ii | stage ii |
| Tumor | TCGA-CV-7255-01 | 1 | 64 | female | 32 | stage iva | stage ii |
| Tumor | TCGA-CV-7261-01 | 0 | 1512 | male | 57 | stage iva | stage iii |
| Tumor | TCGA-CV-7406-01 | 1 | 1748 | male | 49 | stage ii | stage ii |
| Tumor | TCGA-CV-7409-01 | 1 | 543 | male | 43 | stage ivb | stage ivb |
| Tumor | TCGA-CV-7410-01 | 1 | 6417 | male | 61 | stage iii | stage ii |
| Tumor | TCGA-CV-7413-01 | 1 | 294 | female | 74 | stage ii | stage ii |
| Tumor | TCGA-CV-7414-01 | 1 | 14 | male | 78 | stage iva | stage iii |
| Tumor | TCGA-CV-7415-01 | 1 | 695 | male | 60 | stage iva | stage iii |
| Tumor | TCGA-CV-7416-01 | 1 | 763 | female | 29 | stage iva | stage iva |
| Tumor | TCGA-CV-7421-01 | 1 | 2 | male | 76 | stage iva | stage iva |
| Tumor | TCGA-CV-7422-01 | 1 | 1037 | female | 60 | stage iva | stage iva |
| Tumor | TCGA-CV-7424-01 | 1 | 453 | male | 67 | stage iva | stage iva |
| Tumor | TCGA-CV-7429-01 | 1 | 107 | male | 55 | stage iva | stage iii |
| Tumor | TCGA-CV-7430-01 | 1 | 495 | male | 56 | stage iva | stage iii |
| Tumor | TCGA-CV-7433-01 | 1 | 601 | male | 49 | stage iva | stage iva |
| Tumor | TCGA-CV-7435-01 | 1 | 4680 | female | 57 | stage iva | stage iva |
| Tumor | TCGA-CV-7437-01 | 1 | 506 | male | 77 | stage ii | stage ii |
| Tumor | TCGA-CV-7440-01 | 1 | 675 | male | 38 | stage ii | stage ii |
| Tumor | TCGA-CV-7446-01 | 1 | 1093 | male | 66 | stage iva | stage ii |
| Tumor | TCGA-CV-7568-01 | 1 | 927 | female | 48 | stage iva | stage iva |
| Tumor | TCGA-CV-A45R-01 | 0 | 5480 | male | 46 | stage iii | stage iii |
| Tumor | TCGA-CV-A45U-01 | 1 | 1079 | male | 59 | stage iva | stage iva |
| Tumor | TCGA-CV-A45Z-01 | 1 | 1466 | male | 75 | stage ii | stage ii |
| Tumor | TCGA-CV-A460-01 | 1 | 1838 | male | 72 | stage ii | stage iva |
| Tumor | TCGA-CV-A464-01 | 0 | 1722 | male | 48 | stage iii | stage iva |
| Tumor | TCGA-CV-A6JU-01 | 0 | 110 | female | 61 | stage ivb | stage iva |
| Tumor | TCGA-CV-A6K0-01 | 0 | 606 | male | 58 | stage i | stage ii |
| Tumor | TCGA-CV-A6K2-01 | 1 | 317 | male | 79 | stage iva | stage ii |
| Tumor | TCGA-CX-7082-01 | 1 | 11 | male | 82 | stage iva | stage ii |
| Tumor | TCGA-CX-7085-01 | 0 | 321 | female | 77 | stage i | stage iii |
| Tumor | TCGA-CX-7086-01 | 0 | 573 | male | 53 | stage iii | stage iva |
| Tumor | TCGA-CX-A4AQ-01 | 0 | 1555 | male | 56 | stage iva | stage iva |
| Tumor | TCGA-D6-6515-01 | 1 | 403 | female | 82 | stage ii | stage ii |
| Tumor | TCGA-D6-6824-01 | 0 | 77 | male | 61 | stage iva | stage iva |
| Tumor | TCGA-D6-8569-01 | 0 | 770 | male | 52 | stage ii | stage ii |
| Tumor | TCGA-D6-A6EO-01 | 0 | 759 | male | 44 | stage iva | stage iva |
| Tumor | TCGA-D6-A6EP-01 | 0 | 424 | male | 62 | stage iii | stage iii |
| Tumor | TCGA-D6-A74Q-01 | 0 | 710 | male | 67 | stage iva | stage iva |
| Tumor | TCGA-F7-7848-01 | 0 | 1131 | male | 47 | stage iva | stage iva |
| Tumor | TCGA-F7-8298-01 | 0 | 995 | male | 58 | stage i | stage i |
| Tumor | TCGA-H7-8501-01 | 0 | 461 | male | 54 | stage iva | stage iva |
| Tumor | TCGA-HD-7753-01 | 0 | 866 | male | 62 | stage i | stage ii |
| Tumor | TCGA-HD-7754-01 | 0 | 783 | male | 69 | stage iva | stage iva |
| Tumor | TCGA-HD-7831-01 | 0 | 667 | male | 74 | stage iva | stage iii |
| Tumor | TCGA-HD-7917-01 | 1 | 836 | male | 62 | stage ii | stage ii |
| Tumor | TCGA-HD-8224-01 | 1 | 446 | male | 63 | stage iva | stage iii |
| Tumor | TCGA-HD-A634-01 | 1 | 130 | male | 56 | stage iva | stage iii |
| Tumor | TCGA-HD-A6HZ-01 | 0 | 111 | female | 79 | stage iii | stage ii |
| Tumor | TCGA-IQ-7630-01 | 0 | 485 | male | 49 | stage ii | stage iii |
| Tumor | TCGA-IQ-7632-01 | 0 | 441 | female | 68 | stage iva | stage iva |
| Tumor | TCGA-IQ-A61E-01 | 0 | 1147 | female | 55 | stage iii | stage iii |
| Tumor | TCGA-IQ-A61G-01 | 0 | 360 | male | 57 | stage iva | stage iva |
| Tumor | TCGA-IQ-A61H-01 | 0 | 1138 | male | 76 | stage ii | stage ii |
| Tumor | TCGA-IQ-A61O-01 | 1 | 421 | male | 43 | stage iva | stage iva |
| Tumor | TCGA-KU-A66S-01 | 1 | 406 | female | 69 | stage iii | stage iii |
| Tumor | TCGA-KU-A66T-01 | 0 | 552 | female | 53 | stage iva | stage iva |
| Tumor | TCGA-KU-A6H8-01 | 1 | 327 | male | 41 | stage iva | stage i |
| Tumor | TCGA-MT-A7BN-01 | 0 | 469 | male | 74 | stage iva | stage iva |
| Tumor | TCGA-MZ-A7D7-01 | 0 | 547 | male | 51 | stage iva | stage iva |
| Tumor | TCGA-P3-A5Q5-01 | 0 | 910 | male | 54 | stage iva | stage iva |
| Tumor | TCGA-P3-A5QE-01 | 0 | 1559 | male | 49 | stage iii | stage iii |
| Tumor | TCGA-P3-A5QF-01 | 1 | 330 | male | 49 | stage iva | stage iva |
| Tumor | TCGA-P3-A6T0-01 | 0 | 578 | female | 47 | stage iva | stage iva |
| Tumor | TCGA-P3-A6T4-01 | 1 | 62 | male | 54 | stage iva | stage iva |
| Tumor | TCGA-P3-A6T6-01 | 1 | 395 | male | 53 | stage iva | stage iva |
| Tumor | TCGA-P3-A6T8-01 | 0 | 400 | male | 54 | stage iva | stage iva |
| Tumor | TCGA-QK-A6IG-01 | 1 | 222 | male | 69 | stage iii | stage ii |
| Tumor | TCGA-QK-A6VB-01 | 0 | 641 | male | 66 | stage iva | stage iva |
| Tumor | TCGA-QK-A6VC-01 | 0 | 600 | female | 62 | stage iva | stage iva |
| Tumor | TCGA-QK-A8Z7-01 | 0 | 392 | male | 59 | stage iva | stage iva |
| Tumor | TCGA-QK-A8Z8-01 | 1 | 171 | female | 60 | stage ivc | stage ivc |
| Tumor | TCGA-QK-A8Z9-01 | 1 | 449 | male | 56 | stage iva | stage iva |
| Tumor | TCGA-QK-AA3J-01 | 0 | 466 | male | 69 | stage i | stage i |
| Tumor | TCGA-RS-A6TP-01 | 0 | 516 | male | 58 | stage ii | stage ii |
| Tumor | TCGA-T3-A92M-01 | 0 | 417 | male | 52 | stage iva | stage iva |
| Tumor | TCGA-UF-A719-01 | 0 | 1663 | male | 54 | stage ii | stage iii |
| Tumor | TCGA-UF-A71D-01 | 0 | 1461 | female | 54 | stage iva | stage iva |
| Tumor | TCGA-UF-A7J9-01 | 0 | 1358 | male | 75 | stage iva | stage iva |
| Tumor | TCGA-UF-A7JA-01 | 0 | 2265 | female | 66 | stage iva | stage iva |
| Tumor | TCGA-UF-A7JD-01 | 1 | 739 | male | 71 | stage iva | stage iva |
| Tumor | TCGA-UF-A7JJ-01 | 0 | 549 | male | 68 | stage iva | stage iva |
| Tumor | TCGA-UF-A7JK-01 | 1 | 424 | male | 59 | stage iva | stage iva |
| Tumor | TCGA-WA-A7H4-01 | 0 | 443 | male | 69 | stage ii | stage ii |

**Supplementary Table 5. The clinical and demographic characteristics of GSE41613**

| Samples | Sex | Age | Tumor stage | Tissue type | Survival time | Cause of death | Status |
| --- | --- | --- | --- | --- | --- | --- | --- |
| GSM1020099 | F; | 40-49; | I/II; | HPV-negative oral squamous cell carcinoma (OSCC); | 62.85; | Alive; | 0 |
| GSM1020100 | M; | 50-59; | I/II; | HPV-negative oral squamous cell carcinoma (OSCC); | 55.66; | Dead-oral ca; | 1 |
| GSM1020101 | M; | 50-59; | III/IV; | HPV-negative oral squamous cell carcinoma (OSCC); | 6.54; | Dead-oral ca; | 1 |
| GSM1020102 | M; | 60-88; | III/IV; | HPV-negative oral squamous cell carcinoma (OSCC); | 3.38; | Dead-non OC; | 1 |
| GSM1020103 | M; | 40-49; | I/II; | HPV-negative oral squamous cell carcinoma (OSCC); | 28.62; | Dead-oral ca; | 1 |
| GSM1020104 | F; | 40-49; | III/IV; | HPV-negative oral squamous cell carcinoma (OSCC); | 85.03; | Alive; | 0 |
| GSM1020105 | F; | 60-88; | III/IV; | HPV-negative oral squamous cell carcinoma (OSCC); | 40.71; | Dead-oral ca; | 1 |
| GSM1020106 | F; | 60-88; | I/II; | HPV-negative oral squamous cell carcinoma (OSCC); | 84.24; | Alive; | 0 |
| GSM1020107 | M; | 50-59; | III/IV; | HPV-negative oral squamous cell carcinoma (OSCC); | 46.88; | Dead-oral ca; | 1 |
| GSM1020108 | F; | 60-88; | III/IV; | HPV-negative oral squamous cell carcinoma (OSCC); | 82.92; | Alive; | 0 |
| GSM1020109 | M; | 60-88; | III/IV; | HPV-negative oral squamous cell carcinoma (OSCC); | 14.16; | Dead-oral ca; | 1 |
| GSM1020110 | M; | 40-49; | III/IV; | HPV-negative oral squamous cell carcinoma (OSCC); | 10.05; | Dead-oral ca; | 1 |
| GSM1020111 | M; | 50-59; | III/IV; | HPV-negative oral squamous cell carcinoma (OSCC); | 28.52; | Dead-non OC; | 1 |
| GSM1020112 | M; | 60-88; | I/II; | HPV-negative oral squamous cell carcinoma (OSCC); | 81.51; | Alive; | 0 |
| GSM1020113 | M; | 50-59; | III/IV; | HPV-negative oral squamous cell carcinoma (OSCC); | 7.16; | Dead-oral ca; | 1 |
| GSM1020114 | M; | 60-88; | I/II; | HPV-negative oral squamous cell carcinoma (OSCC); | 7.23; | Dead-oral ca; | 1 |
| GSM1020115 | M; | 50-59; | III/IV; | HPV-negative oral squamous cell carcinoma (OSCC); | 66.63; | Dead-non OC; | 1 |
| GSM1020116 | M; | 40-49; | III/IV; | HPV-negative oral squamous cell carcinoma (OSCC); | 78.29; | Dead -unk cause; | 1 |
| GSM1020117 | M; | 50-59; | III/IV; | HPV-negative oral squamous cell carcinoma (OSCC); | 9.92; | Dead -unk cause; | 1 |
| GSM1020118 | F; | 40-49; | III/IV; | HPV-negative oral squamous cell carcinoma (OSCC); | 15.31; | Dead-oral ca; | 1 |
| GSM1020119 | M; | 50-59; | III/IV; | HPV-negative oral squamous cell carcinoma (OSCC); | 80.16; | Alive; | 0 |
| GSM1020120 | M; | 40-49; | I/II; | HPV-negative oral squamous cell carcinoma (OSCC); | 80.79; | Alive; | 0 |
| GSM1020121 | F; | 40-49; | III/IV; | HPV-negative oral squamous cell carcinoma (OSCC); | 15.41; | Dead-oral ca; | 1 |
| GSM1020122 | F; | 50-59; | I/II; | HPV-negative oral squamous cell carcinoma (OSCC); | 18.40; | Dead-non OC; | 1 |
| GSM1020123 | M; | 40-49; | III/IV; | HPV-negative oral squamous cell carcinoma (OSCC); | 21.78; | Dead-oral ca; | 1 |
| GSM1020124 | F; | 60-88; | III/IV; | HPV-negative oral squamous cell carcinoma (OSCC); | 24.25; | Dead-oral ca; | 1 |
| GSM1020125 | F; | 19-39; | III/IV; | HPV-negative oral squamous cell carcinoma (OSCC); | 5.85; | Dead-oral ca; | 1 |
| GSM1020126 | F; | 60-88; | I/II; | HPV-negative oral squamous cell carcinoma (OSCC); | 35.45; | Dead -unk cause; | 1 |
| GSM1020127 | M; | 60-88; | III/IV; | HPV-negative oral squamous cell carcinoma (OSCC); | 78.95; | Alive; | 0 |
| GSM1020128 | F; | 50-59; | III/IV; | HPV-negative oral squamous cell carcinoma (OSCC); | 41.17; | Dead-non OC; | 1 |
| GSM1020129 | F; | 60-88; | III/IV; | HPV-negative oral squamous cell carcinoma (OSCC); | 76.75; | Dead -unk cause; | 1 |
| GSM1020130 | M; | 60-88; | III/IV; | HPV-negative oral squamous cell carcinoma (OSCC); | 3.52; | Dead-oral ca; | 1 |
| GSM1020131 | F; | 60-88; | III/IV; | HPV-negative oral squamous cell carcinoma (OSCC); | 24.87; | Dead-oral ca; | 1 |
| GSM1020132 | M; | 50-59; | III/IV; | HPV-negative oral squamous cell carcinoma (OSCC); | 20.11; | Dead-oral ca; | 1 |
| GSM1020133 | M; | 60-88; | I/II; | HPV-negative oral squamous cell carcinoma (OSCC); | 76.85; | Alive; | 0 |
| GSM1020134 | M; | 40-49; | I/II; | HPV-negative oral squamous cell carcinoma (OSCC); | 29.14; | Dead-oral ca; | 1 |
| GSM1020135 | F; | 50-59; | III/IV; | HPV-negative oral squamous cell carcinoma (OSCC); | 26.81; | Dead-non OC; | 1 |
| GSM1020136 | M; | 60-88; | I/II; | HPV-negative oral squamous cell carcinoma (OSCC); | 75.07; | Alive; | 0 |
| GSM1020137 | M; | 60-88; | III/IV; | HPV-negative oral squamous cell carcinoma (OSCC); | 43.24; | Dead-non OC; | 1 |
| GSM1020138 | F; | 50-59; | I/II; | HPV-negative oral squamous cell carcinoma (OSCC); | 74.64; | Alive; | 0 |
| GSM1020139 | M; | 19-39; | III/IV; | HPV-negative oral squamous cell carcinoma (OSCC); | 14.75; | Dead-oral ca; | 1 |
| GSM1020140 | M; | 60-88; | I/II; | HPV-negative oral squamous cell carcinoma (OSCC); | 73.17; | Alive; | 0 |
| GSM1020141 | M; | 60-88; | III/IV; | HPV-negative oral squamous cell carcinoma (OSCC); | 72.28; | Alive; | 0 |
| GSM1020142 | M; | 50-59; | III/IV; | HPV-negative oral squamous cell carcinoma (OSCC); | 73.26; | Alive; | 0 |
| GSM1020143 | M; | 50-59; | I/II; | HPV-negative oral squamous cell carcinoma (OSCC); | 64.99; | Dead -unk cause; | 1 |
| GSM1020144 | F; | 60-88; | III/IV; | HPV-negative oral squamous cell carcinoma (OSCC); | 68.47; | Alive; | 0 |
| GSM1020145 | M; | 60-88; | III/IV; | HPV-negative oral squamous cell carcinoma (OSCC); | 12.29; | Dead-oral ca; | 1 |
| GSM1020146 | F; | 60-88; | III/IV; | HPV-negative oral squamous cell carcinoma (OSCC); | 6.44; | Dead-oral ca; | 1 |
| GSM1020147 | F; | 50-59; | I/II; | HPV-negative oral squamous cell carcinoma (OSCC); | 69.16; | Alive; | 0 |
| GSM1020148 | F; | 19-39; | I/II; | HPV-negative oral squamous cell carcinoma (OSCC); | 70.08; | Alive; | 0 |
| GSM1020149 | F; | 50-59; | I/II; | HPV-negative oral squamous cell carcinoma (OSCC); | 70.08; | Alive; | 0 |
| GSM1020150 | M; | 60-88; | III/IV; | HPV-negative oral squamous cell carcinoma (OSCC); | 68.14; | Alive; | 0 |
| GSM1020151 | F; | 60-88; | I/II; | HPV-negative oral squamous cell carcinoma (OSCC); | 67.94; | Alive; | 0 |
| GSM1020152 | M; | 50-59; | I/II; | HPV-negative oral squamous cell carcinoma (OSCC); | 19.65; | Dead-non OC; | 1 |
| GSM1020153 | M; | 19-39; | III/IV; | HPV-negative oral squamous cell carcinoma (OSCC); | 67.55; | Alive; | 0 |
| GSM1020154 | M; | 50-59; | I/II; | HPV-negative oral squamous cell carcinoma (OSCC); | 66.60; | Alive; | 0 |
| GSM1020155 | M; | 19-39; | I/II; | HPV-negative oral squamous cell carcinoma (OSCC); | 66.56; | Alive; | 0 |
| GSM1020156 | M; | 60-88; | I/II; | HPV-negative oral squamous cell carcinoma (OSCC); | 65.22; | Alive; | 0 |
| GSM1020157 | M; | 60-88; | III/IV; | HPV-negative oral squamous cell carcinoma (OSCC); | 2.40; | Dead-oral ca; | 1 |
| GSM1020158 | M; | 50-59; | III/IV; | HPV-negative oral squamous cell carcinoma (OSCC); | 10.68; | Dead-oral ca; | 1 |
| GSM1020159 | F; | 40-49; | III/IV; | HPV-negative oral squamous cell carcinoma (OSCC); | 65.45; | Alive; | 0 |
| GSM1020160 | M; | 60-88; | I/II; | HPV-negative oral squamous cell carcinoma (OSCC); | 63.54; | Alive; | 0 |
| GSM1020161 | M; | 50-59; | I/II; | HPV-negative oral squamous cell carcinoma (OSCC); | 63.38; | Alive; | 0 |
| GSM1020162 | F; | 60-88; | III/IV; | HPV-negative oral squamous cell carcinoma (OSCC); | 35.12; | Dead-oral ca; | 1 |
| GSM1020163 | M; | 60-88; | III/IV; | HPV-negative oral squamous cell carcinoma (OSCC); | 60.55; | Alive; | 0 |
| GSM1020164 | M; | 60-88; | III/IV; | HPV-negative oral squamous cell carcinoma (OSCC); | 61.54; | Alive; | 0 |
| GSM1020165 | M; | 60-88; | III/IV; | HPV-negative oral squamous cell carcinoma (OSCC); | 19.12; | Dead-oral ca; | 1 |
| GSM1020166 | M; | 60-88; | I/II; | HPV-negative oral squamous cell carcinoma (OSCC); | 61.73; | Alive; | 0 |
| GSM1020167 | F; | 50-59; | III/IV; | HPV-negative oral squamous cell carcinoma (OSCC); | 51.61; | Dead -unk cause; | 1 |
| GSM1020168 | M; | 40-49; | III/IV; | HPV-negative oral squamous cell carcinoma (OSCC); | 61.08; | Alive; | 0 |
| GSM1020169 | M; | 50-59; | III/IV; | HPV-negative oral squamous cell carcinoma (OSCC); | 59.47; | Alive; | 0 |
| GSM1020170 | M; | 60-88; | I/II; | HPV-negative oral squamous cell carcinoma (OSCC); | 61.01; | Alive; | 0 |
| GSM1020171 | M; | 60-88; | III/IV; | HPV-negative oral squamous cell carcinoma (OSCC); | 60.65; | Alive; | 0 |
| GSM1020172 | F; | 60-88; | I/II; | HPV-negative oral squamous cell carcinoma (OSCC); | 59.96; | Alive; | 0 |
| GSM1020173 | M; | 60-88; | I/II; | HPV-negative oral squamous cell carcinoma (OSCC); | 9.86; | Dead-oral ca; | 1 |
| GSM1020174 | M; | 40-49; | III/IV; | HPV-negative oral squamous cell carcinoma (OSCC); | 9.23; | Dead-oral ca; | 1 |
| GSM1020175 | M; | 50-59; | I/II; | HPV-negative oral squamous cell carcinoma (OSCC); | 59.24; | Alive; | 0 |
| GSM1020176 | M; | 60-88; | III/IV; | HPV-negative oral squamous cell carcinoma (OSCC); | 6.90; | Dead-oral ca; | 1 |
| GSM1020177 | F; | 60-88; | I/II; | HPV-negative oral squamous cell carcinoma (OSCC); | 58.71; | Alive; | 0 |
| GSM1020178 | F; | 60-88; | III/IV; | HPV-negative oral squamous cell carcinoma (OSCC); | 1.22; | Dead-non OC; | 1 |
| GSM1020179 | M; | 60-88; | I/II; | HPV-negative oral squamous cell carcinoma (OSCC); | 62.85; | Alive; | 0 |
| GSM1020180 | M; | 40-49; | III/IV; | HPV-negative oral squamous cell carcinoma (OSCC); | 57.63; | Alive; | 0 |
| GSM1020181 | M; | 50-59; | III/IV; | HPV-negative oral squamous cell carcinoma (OSCC); | 45.93; | Dead-non OC; | 1 |
| GSM1020182 | M; | 50-59; | III/IV; | HPV-negative oral squamous cell carcinoma (OSCC); | 17.02; | Dead-non OC; | 1 |
| GSM1020183 | M; | 60-88; | I/II; | HPV-negative oral squamous cell carcinoma (OSCC); | 35.38; | Dead-non OC; | 1 |
| GSM1020184 | M; | 50-59; | I/II; | HPV-negative oral squamous cell carcinoma (OSCC); | 14.42; | Dead-non OC; | 1 |
| GSM1020185 | M; | 60-88; | III/IV; | HPV-negative oral squamous cell carcinoma (OSCC); | 6.54; | Dead-oral ca; | 1 |
| GSM1020186 | M; | 40-49; | I/II; | HPV-negative oral squamous cell carcinoma (OSCC); | 57.53; | Alive; | 0 |
| GSM1020187 | M; | 60-88; | III/IV; | HPV-negative oral squamous cell carcinoma (OSCC); | 0.46; | Dead-non OC; | 1 |
| GSM1020188 | M; | 60-88; | I/II; | HPV-negative oral squamous cell carcinoma (OSCC); | 56.02; | Alive; | 0 |
| GSM1020189 | M; | 60-88; | I/II; | HPV-negative oral squamous cell carcinoma (OSCC); | 55.10; | Alive; | 0 |
| GSM1020190 | M; | 50-59; | III/IV; | HPV-negative oral squamous cell carcinoma (OSCC); | 20.01; | Dead -unk cause; | 1 |
| GSM1020191 | F; | 19-39; | III/IV; | HPV-negative oral squamous cell carcinoma (OSCC); | 8.38; | Dead-oral ca; | 1 |
| GSM1020192 | M; | 40-49; | I/II; | HPV-negative oral squamous cell carcinoma (OSCC); | 54.57; | Alive; | 0 |
| GSM1020193 | F; | 60-88; | I/II; | HPV-negative oral squamous cell carcinoma (OSCC); | 54.41; | Alive; | 0 |
| GSM1020194 | F; | 60-88; | I/II; | HPV-negative oral squamous cell carcinoma (OSCC); | 53.88; | Alive; | 0 |
| GSM1020195 | M; | 60-88; | I/II; | HPV-negative oral squamous cell carcinoma (OSCC); | 52.60; | Alive; | 0 |

**Supplementary Table 6.** **The clinical and demographic characteristics of GSE42743**

| Smaples | Age | Gender | Smoking status | T stage | Local recurrence | Survival time | Status |
| --- | --- | --- | --- | --- | --- | --- | --- |
| GSM1049079 | 54; | Male; | Current; | 4; | 0; | 359; | 1; |
| GSM1049080 | 42; | Male; | Current; | 2; | 0; | 50; | 0; |
| GSM1049081 | 28; | Male; | Former; | 3; | 0; | 1042; | 0; |
| GSM1049082 | 75; | Male; | Current; | 3; | 0; | 93; | 1; |
| GSM1049083 | 59; | Male; | Current; | 4; | 0; | 670; | 0; |
| GSM1049084 | 73; | Male; | Current; | 3; | 0; | 167; | 1; |
| GSM1049085 | 84; | Male; | NeverSmoker; | 3; | 0; | 338; | 1; |
| GSM1049086 | 61; | Male; | Former; | 2; | 0; | 163; | 1; |
| GSM1049087 | 73; | Female; | NeverSmoker; | 2; | 0; | 405; | 1; |
| GSM1049088 | 60; | Female; | NeverSmoker; | 2; | 0; | 413; | 1; |
| GSM1049089 | 22; | Male; | NeverSmoker; | 2; | 1; | 226; | 1; |
| GSM1049090 | 68; | Male; | Former; | 4; | 0; | 157; | 1; |
| GSM1049091 | 45; | Male; | Current; | 3; | 0; | 608; | 1; |
| GSM1049092 | 60; | Male; | Current; | 4; | 0; | 1540; | 0; |
| GSM1049093 | 75; | Male; | Former; | 3; | 0; | 865; | 0; |
| GSM1049094 | 64; | Male; | NeverSmoker; | 3; | 0; | 791; | 0; |
| GSM1049095 | 67; | Male; | Current; | 2; | 0; | 308; | 0; |
| GSM1049096 | 46; | Female; | Current; | 4; | 0; | 516; | 1; |
| GSM1049097 | 65; | Male; | Former; | 2; | 0; | 142; | 1; |
| GSM1049098 | 57; | Male; | Current; | 3; | 0; | 890; | 0; |
| GSM1049099 | 48; | Male; | Current; | 3; | 0; | 312; | 0; |
| GSM1049100 | 39; | Male; | Former; | 3; | 0; | 352; | 1; |
| GSM1049101 | 38; | Female; | NeverSmoker; | 3; | 1; | 239; | 1; |
| GSM1049102 | 62; | Female; | NeverSmoker; | 4; | 0; | 293; | 0; |
| GSM1049103 | 60; | Male; | Current; | 2; | 0; | 623; | 0; |
| GSM1049104 | 47; | Female; | Current; | 2; | 1; | 1060; | 1; |
| GSM1049105 | 70; | Male; | Former; | 3; | 0; | 927; | 0; |
| GSM1049107 | 62; | Male; | Former; | 2; | 0; | 35; | 1; |
| GSM1049109 | 58; | Male; | NeverSmoker; | 2; | 0; | 778; | 1; |
| GSM1049111 | 75; | Male; | Former; | 2; | ?; | 162; | 1; |
| GSM1049114 | 44; | Female; | Former; | 2; | 0; | 128; | 0; |
| GSM1049116 | 75; | Male; | Former; | 3; | 0; | 753; | 0; |
| GSM1049117 | 49; | Male; | Current; | 2; | 0; | 1589; | 1; |
| GSM1049119 | 54; | Male; | Former; | 2; | 0; | 638; | 0; |
| GSM1049120 | 28; | Male; | Current; | 3; | 1; | 239; | 1; |
| GSM1049121 | 39; | Male; | Former; | 3; | ?; | 26; | 1; |
| GSM1049122 | 50; | Male; | Current; | 2; | 0; | 1658; | 0; |
| GSM1049124 | 41; | Male; | Current; | 1; | 0; | 1032; | 0; |
| GSM1049125 | 62; | Male; | Former; | 2; | 0; | 128; | 1; |
| GSM1049128 | 67; | Female; | Former; | 2; | 0; | 379; | 1; |
| GSM1049129 | 44; | Male; | Former; | 3; | 0; | 884; | 1; |
| GSM1049131 | 62; | Male; | Former; | 2; | ?; | 21; | 1; |
| GSM1049133 | 55; | Male; | Former; | 4; | 0; | 847; | 0; |
| GSM1049134 | 76; | Male; | Former; | 3; | 0; | 26; | 1; |
| GSM1049136 | 48; | Male; | NeverSmoker; | 3; | ?; | 263; | 1; |
| GSM1049137 | 80; | Female; | NeverSmoker; | 3; | 0; | 189; | 1; |
| GSM1049139 | 43; | Male; | Current; | 3; | 0; | 678; | 0; |
| GSM1049141 | 66; | Male; | Current; | 4; | 0; | 556; | 1; |
| GSM1049143 | 58; | Male; | Current; | 3; | 0; | 163; | 1; |
| GSM1049145 | 71; | Male; | Current; | 3; | 1; | 812; | 1; |
| GSM1049146 | 73; | Male; | NeverSmoker; | 1; | 0; | 1479; | 0; |
| GSM1049147 | 57; | Male; | Current; | 3; | 0; | 195; | 1; |
| GSM1049149 | 74; | Female; | NeverSmoker; | 2; | 0; | 890; | 0; |
| GSM1049150 | 41; | Male; | Current; | 3; | 1; | 507; | 0; |
| GSM1049151 | 49; | Male; | Former; | 4; | ?; | 702; | 0; |
| GSM1049155 | 77; | Male; | NeverSmoker; | 3; | ?; | 259; | 1; |
| GSM1049156 | 76; | Male; | NeverSmoker; | 2; | 0; | 1470; | 0; |
| GSM1049158 | 65; | Female; | Current; | 4; | 0; | 122; | 1; |
| GSM1049160 | 71; | Female; | Current; | 3; | 0; | 10; | 1; |
| GSM1049162 | 57; | Female; | Current; | 2; | 0; | 1225; | 0; |
| GSM1049164 | 65; | Male; | Former; | 2; | 0; | 1514; | 0; |
| GSM1049167 | 58; | Female; | Former; | 2; | 0; | 653; | 0; |
| GSM1049169 | 82; | Male; | Former; | 2; | 0; | 658; | 0; |
| GSM1049171 | 63; | Male; | Former; | 4; | ?; | 46; | 1; |
| GSM1049172 | 64; | Female; | Current; | 4; | ?; | 31; | 1; |
| GSM1049173 | 65; | Male; | Former; | 3; | 0; | 2818; | 0; |
| GSM1049174 | 53; | Male; | Former; | 4; | 0; | 2193; | 1; |
| GSM1049175 | 45; | Male; | Current; | 4; | 0; | 470; | 1; |
| GSM1049176 | 59; | Male; | Former; | 2; | 0; | 1680; | 0; |
| GSM1049177 | 38; | Male; | NeverSmoker; | 2; | ?; | 7; | 0; |
| GSM1049178 | 52; | Male; | Former; | 1; | 0; | 1948; | 0; |
| GSM1049179 | 44; | Male; | Former; | 4; | 0; | 96; | 1; |
| GSM1049180 | 59; | Male; | Current; | 3; | 0; | 173; | 1; |
| GSM1049181 | 65; | Female; | Former; | 4; | 0; | 885; | 1; |

**Supplementary Table 7. Univariate and multivariate survival analyses (proportional hazards method) of LSD1 and JMJD3 expression in prognostic prediction for patients from GSE41613.**

| **Variable** | **Univariate survival analysis** | | | **Multivariate survival analysis** | | |
| --- | --- | --- | --- | --- | --- | --- |
|  | **Hazard ratio** | **95% CI** | ***P*-value** | **Hazard ratio** | **95% CI** | ***P*-value** |
| Gender (male, female) | 1.122 | 0.621-2.029 | 0.703 | 0.889 | 0.471-1.681 | 0.718 |
| Age (≤60, >60) | 0.739 | 0.423-1.292 | 0.289 | 0.950 | 0.532-1.696 | 0.861 |
| Tumor stage (I-II, III-IV) | 3.828 | 1.958-7.482 | **<0.001** | 3.875 | 1.969-7.624 | **<0.001** |
| LSD1/JMJD3 |  |  |  |  |  |  |
| LSD1^low^JMJD3^low^ | 1 | 1 | **0.046** | 1 | 1 | 0.058 |
| LSD1^low^JMJD3^high^ | 1.771 | 0.697-4.501 | 0.230 | 2.076 | 0.773-5.577 | 0.147 |
| LSD1^high^JMJD3^low^ | 1.968 | 0.554-6.985 | 0.295 | 2.431 | 0.674-8.770 | 0.175 |
| LSD1^high^JMJD3^high^ | 3.231 | 1.314-7.947 | **0.011** | 3.472 | 1.353-8.911 | **0.010** |

**The numbers in bold indicate statistical significance with *P*-values less than 0.05.**
